# The utility of algae as sources of high value nutritional ingredients, particularly for alternative/complementary proteins to improve human health

**DOI:** 10.3389/fnut.2023.1277343

**Published:** 2023-10-13

**Authors:** Jia Yee Wu, Rachel Tso, Hwee Sze Teo, Sumanto Haldar

**Affiliations:** ^1^Clinical Nutrition Research Centre, Singapore Institute of Food and Biotechnology Innovation, Agency for Science, Technology and Research (A*STAR), Singapore, Singapore; ^2^Faculty of Health and Social Sciences, Bournemouth University, Bournemouth, United Kingdom

**Keywords:** seaweed, algae, microalgae, alternative protein, nutrition, metabolic health, food applications, protein quality

## Abstract

As the global population continues to grow, the demand for dietary protein is rapidly increasing, necessitating the exploration of sustainable and nutritious protein sources. Algae has emerged as a promising food source due to their high value ingredients such as proteins, as well as for their environmental sustainability and abundance. However, knowledge gaps surrounding dietary recommendations and food applications restrict algae’s utilization as a viable protein source. This review aims to address these gaps by assessing the suitability of both microalgae and macroalgae as alternative/complementary protein sources and exploring their potential applications in food products. The first section examines the potential suitability of algae as a major food source by analyzing the composition and bioavailability of key components in algal biomass, including proteins, lipids, dietary fiber, and micronutrients. Secondly, the biological effects of algae, particularly their impact on metabolic health are investigated with an emphasis on available clinical evidence. While evidence reveals protective effects of algae on glucose and lipid homeostasis as well as anti-inflammatory properties, further research is required to understand the longer-term impact of consuming algal protein, protein isolates, and concentrates on metabolic health, including protein metabolism. The review then explores the potential of algal proteins in food applications, including ways to overcome their sensory limitations, such as their dark pigmentation, taste, and odor, in order to improve consumer acceptance. To maximize algae’s potential as a valuable protein source in the food sector, future research should prioritize the production of more acceptable algal biomass and explore new advances in food sciences and technology for improved consumer acceptance. Overall, this paper supports the potential utility of algae as a sustainable and healthy ingredient source for widespread use in future food production.

## 1. Introduction

It is projected that in 2050, the world’s population will reach 10 billion people (1, 2), necessitating a 35 to 56% increase in food production from 2010 to 2050 (1). Concurrently, due to shifts in dietary preferences toward protein-rich food consumption, demand for dietary protein continues to grow. Adding another level of complexity to this issue is the consideration of reducing environmental burdens while improving global nutrition, as outlined by the United Nations’ Sustainable Development Goals (3). In light of these factors, the exploration of alternative and complementary protein sources that are sustainable, nutritious, and affordable becomes crucial (4, 5).

Algae are a diverse group of photosynthetic eukaryotes. They encompass both microalgae, which are microscopic and unicellular, and complex macroalgae, which are commonly known as seaweed (6). Algae has gained attention as a potential solution to this increasing demand due to its sustainability advantages, excellent nutritional profile, and opportunities in food applications as well as nutraceuticals (7). Compared to most terrestrial plants, algae have a higher growth rate, productivity, and protein yield, making it a more sustainable source of protein (8). Algae cultivation is resource efficient in the already burdened agri-food system (7, 9), since it does not compete with traditional food sources for potable water and arable land (10–12). Algae can be cultivated in a wide range of environments, from oceans and lakes to controlled photobioreactors, either carbon-neutral photoautotrophically, or heterotrophically utilizing minimal carbon sources (8). This makes algae cultivation contribute to a lower environmental footprint compared to conventional protein sources (7, 13, 14). As part of the marine circular economy approach (12), algae’s potential in mitigating greenhouse gas emissions while generating high value organic nutrients or biofuel through CO_2_ biofixation (15–17) and industrial CO_2_ trapping (18) has been explored. Therefore, algae’s versatility extends beyond food production, making it a highly attractive alternative to conventional protein sources.

Despite the growing interest in algae, its widespread adoption in the food industry is still in its preliminary stages. While algae can be cultivated in controlled environments, scaling up production to meet the consistent quality and yield required for mainstream food production poses challenges (19). Additionally, with algae-based food products still being relatively new to the market for a substantial part of the population, consumer understanding and acceptance as well as the regulatory landscape for algae-based food products is still evolving. Limited knowledge regarding the suitability of algae in food applications, particularly from both nutrition and food technology perspectives, presents significant barriers to its integration into mainstream food systems. Similar to the introduction of all novel foods, increasing awareness and knowledge about algae as well as increasing the product range can encourage consumers to embrace new eating habits that include algae in their diets, which can ultimately lead to increased demand, economies of scale, and accessibility of algae products to a wider population. Taste, cost, and nutrition are essential characteristics of a successful food product, all of which impact consumer preferences and influence purchasing decisions. Algae has promising prospects in satisfying all three, which will be addressed in terms of algae’s nutritional features, followed by its sensory properties, and finally its prospective food uses.

While numerous studies have recognized algae’s potential to provide essential or high value nutrients such as fatty acids, minerals, or dietary fiber, this review aims to critically assess the suitability of algae to be consumed as a regular protein source. This work focuses on edible species of macroalgae and microalgae and considers their nutritional compositions, nutrient bioavailability, and accompanied health impacts based on clinical evidence. The second part of the review will explore the opportunities of incorporating algae in food applications and summarize the outcomes of previous attempts of algal food applications. Consumer acceptance and potential challenges will be examined to identify the sweet spot for successful algae incorporation in food products. Finally, future research directions will be discussed to address the identified challenges and prioritize further advancements in algae utilization in the food industry.

## 2. Edible algae

Despite being classified as a novel food, algae have been harvested and consumed by coastal populations for centuries, dating back to prehistoric times (20). The long-standing tradition of their consumption has been reviewed elsewhere (21, 22), highlighting their significance as an integral part of traditional diets and culinary practices, particularly in Latin American and Asian cultures.

**Figure 1:**
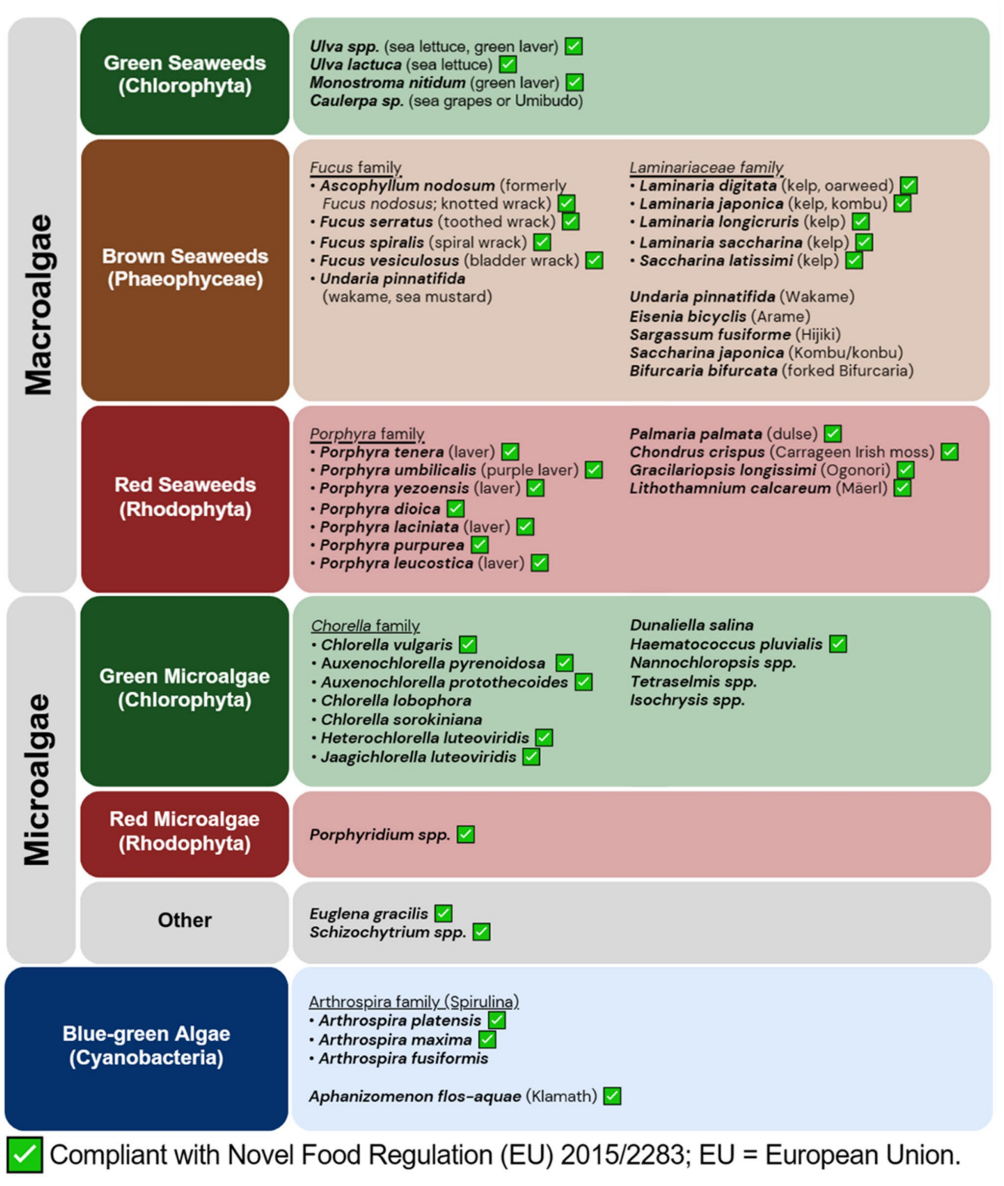
Overview of edible algae and their regulatory compliance. Common edible algae are broadly categorized according to their pigments. Cyanobacteria is included even though they are not considered as true algae. A green checkbox indicates compliance with the European Union’s Novel Food Regulation (EU 2015/2283). Data accurate as of June 20, 2023.

While the phylogeny relationship of algae is continuing to be updated as new ones are discovered, algae is broadly classified into three groups depending on their pigments, namely green (chlorophytes), brown (phaeophytes), and red (rhodophytes) algae. Although not part of algae, cyanobacteria are often referred to as blue-green algae, which will also be included in this article. Figure 1 illustrates the broad classification of algae based on their pigments.

### 2.1. Macroalgae (seaweeds)

#### 2.1.1. Green seaweeds (chlorophytes)

The green seaweeds (chlorophytes), such as sea lettuce (*Ulva* spp.) and sea grapes (*Caulerpa* spp.), are known for their vibrant green color and high protein content. Sea lettuce (*Ulva* spp.) is commonly used in salads and soups whereas sea grapes or Umibudo (*Caulerpa* spp.) are a popular edible green seaweed in Japan and Southeast Asian countries.

#### 2.1.2. Brown seaweeds (phaeophytes)

Brown seaweeds (phaeophytes) feature varieties such as wakame (*Undaria pinnatifida*; referred hereafter as *U. pinnatifida*), arame (*Eisenia bicyclis*), hijiki (*Sargassum fusiforme*), kombu/konbu (*Saccharina japonica*; referred hereafter as *S. japonica*), and various kelp (*Laminariaceae* family). Wakame is a popular brown seaweed used in Asian cuisine, known for its tender texture and mild flavor. Arame and hijiki are commonly used in Japanese cuisine as well, adding distinct flavors and textures to various dishes. Kelp, with its large size and high concentrations of alginate, fucoxanthin, and fucoidan content, offers a wide range of functional and nutritional properties. Notable edible kelps include cochayuyo and kombu. Kombu is widely used in Japanese cuisine, providing a unique umami flavor.

#### 2.1.3. Red seaweeds (rhodophytes)

Red seaweed (rhodophytes) includes commonly eaten seaweeds such as nori/laver (*Porphyra* spp.), dulse (*Palmaria palmata*; referred hereafter as *P. palmata*), and Irish moss (*Chondrus crispus*; referred hereafter as *C. crispus*). Nori is known for its use in sushi rolls. Dulse, commonly found in cool Artic temperatures, is known for its distinct flavor, and often used in soups, salads, and as a snack. Irish moss, also known as carrageen moss, is frequently used in food goods as a thickening ingredient. Red seaweeds are valued for their unique pigments like phycoerythrin.

### 2.2. Microalgae

Microalgae, on the other hand, are unicellular photosynthetic organisms that offer a range of nutritional benefits and have gained recognition as a dietary supplement. Notable edible species include *Chlorella,* and cyanobacteria (or blue-green algae) such as Spirulina and *Aphanizomenon flos-aquae* (referred hereafter as *A. flos-aquae*). Other candidates that have potential to be commercialized for human consumption are *Tetraselmis*, *Isochrysis* and *Nannochloropsis* (23). Each of these species has unique nutritional profiles and benefits (24) and is commonly consumed in the form of powdered supplements or pills, with recommended daily doses ranging from 1 to 5 grams (25).

Edible *Chlorella* spp. include *C. vulgaris*, *Auxenochlorella pyrenoidosa* (formerly known as *C. protothecoides)* (26), *Auxenochlorella protothecoides* (formerly known as *C. protothecoides*), and *C. lobophora* and *C. sorokiniana* which are green microalgae that possess a rich composition of protein, fat, carbohydrate, fiber, chlorophyll, vitamins, and minerals (27). However, *Chlorella* hard cell wall is indigestible by humans and must be broken down either by mechanical or enzymatic degradation before consumption (28). Once the cell wall is broken, *Chlorella* is typically consumed as a supplement in tablet, capsule, liquid, or powder form.

Commonly known as Spirulina*, Arthrospira* has been consumed by the Aztecs for its endurance-boosting properties. Edible *Arthrospira* spp., such as *A. platensis* and *A. maxima* are only remotely related to the Spirulina genera; yet they are still marketed under the commercial name “Spirulina” (29). It is known as a superfood rich in protein, vitamins, and minerals, making it an ideal dietary supplement for vegetarians or vegans (30).

### 2.3. Regulation of algae-derived novel foods

Concerning the regulatory features of novel foods derived from algae, the European Union mandates compliance with Novel Food Regulation (EU) 2015/2283 (31). To be exempt from the legislation, the product must have been in circulation as a food or food additive with substantial consumption prior to 15 May 1997. If not, a safety assessment of the novel food’s compositional, nutritional, toxicological, and allergenic attributes, as well as information regarding the production process, proposed uses, and dosage levels, is required before it can be approved for distribution on the food market (31). As of 20th June 2023, several microalgae species and ingredients have been approved for use in the European Union. These include various species from the family of *Chlorella* spp., such as *C. vulgaris* and those previously classified as *Chlorella* spp., such as *Auxenchoella pyrenoidosa, Auxenchorella protothecoides*, *Heterochlorella luteoviridis* and *Jaagichlorella luteoviridis*. Other approved microalgae include Spirulina (*A. platensis*), Klamath (*A. flos-aquae*), *Euglena gracilis* (*E. gracilis*), *Haematococcus pluvialis*, *Schizochytrium* spp., and red microalgae *Porphyridium* spp. (32).

For seaweeds, several green, red, and brown seaweed species are listed as approved (32). Approved green seaweeds include aonori or green laver (*Ulva* spp., previously known as *Enteromorpha* spp.) and sea lettuce (*Ulva lactuca*; referred hereafter as *U. lactuca*), and *Monostroma nitidum*. Approved red seaweeds include nori which is a family of *Porphyra* spp., such as *Porphyra tenera, Porphyra umbilicalis, Porphyra yezoensis, Porphyra dioica, Porphyra laciniata, Porphyra purpurea, Porphyra leucostica* (all referred hereafter with the genus name shortened to its first letter). Other approved red seaweeds include dulses (*P. palmata)*, Irish moss or Carrageen moss (*C. crispus*), the agar-producing Ogonori or *Gracilariopsis longissimi*, and the calcium-rich Mäerl (*Lithothamnium calcareum*). Approved brown seaweeds include kelp from the *Laminariaceae* family, such as *Laminaria digitata, Laminaria japonica, Laminaria longicruris, Laminaria saccharina,* and *Saccharina latissimi*, as well as the *Fucus* family, such as *Ascophyllum nodosum, Fucus nodosus, Fucus serratus, Fucus spiralis* and *Fucus vesiculosus* (all referred hereafter with the genus name shortened to its first letter).

## 3. Assessing the suitability of microalgae and macroalgae as sources of alternative/complementary proteins

Algae are an ideal source of nutrients as they are rich in protein, lipids, vitamins, minerals, and essential fatty acids (25). Despite being consumed as a food source, seaweed’s potential to complement conventional protein sources is not widely recognized (33). In contrast, microalgae like Spirulina and *Chlorella* have gained popularity as dietary supplements mainly for their dietary fiber and other minor nutritional components. While the consumption of microalgae as dietary supplements is well-established, there is still much to be discovered and understood regarding their utilization as a primary protein source in the form of food (23, 33).

Section 3 will evaluate the suitability of algae as an alternative/complementary source of protein. This entails analyzing the major constituents of algae, including their macronutrient composition and bioavailability. Particularly, this section covers in-depth analyses that evaluate protein quality, amino acid composition, and briefly provides an overview of the presence of other nutrients such as carbohydrates/fiber, lipids, vitamins, and minerals as well as bioactive phytonutrients such as polyphenols and carotenoids. Furthermore, we will investigate the effects of algal consumption on digestion, metabolism, and overall health.

### 3.1. Overview of the nutritional composition of algae focusing on protein content and functionality

The nutritional composition and cellular structure of algae may vary depending on the strain, geographical region, and season, as well as the strain’s response to various factors such as light intensity, photoperiod, temperature, available nutrients, and growth phase (22, 34, 35).

In general, algae are abundant in dietary fiber, ω-3 and ω-6 fatty acids, vitamins (including A, C, D, E, K, and B vitamins), and essential minerals such as iron, calcium, magnesium, and potassium. Microalgae are abundant in protein, with species such as *C. vulgaris*, *C. pyrenoidosa*, *A platensis*, *A. maxima* containing 50–70% protein by dry weight, which comprises all nine essential amino acids (EAA) (25, 34). This makes algae valuable protein sources equivalent to those derived from animals (25, 34).

In addition to their rich nutritional profiles, algae contain primary metabolites such as polysaccharides and phycobiliproteins that may enhance the rheological and nutritional qualities of food. In addition, algae contains secondary metabolites including polyphenols and xanthophylls, which possess antioxidant and other bioactive properties (36). More details on the nutritional composition of algae are elaborated below.

#### 3.1.1. Estimation of algal protein content

According to review articles published over the last 20 years, red seaweed is a rich source of protein, with laver *Porphyra tenera* containing up to 47% protein (37–42). However, tracking the origin of this high number revealed that it was based upon a study conducted in 1983 (43). Another study conducted by Admassu et al. (44) was able to corroborate a similar finding for red seaweed laver, albeit with a slightly lower (43%) reported percentage. However, both studies employed a nitrogen-to-protein conversion rate of 6.25 (44, 45), which results in an overestimation of protein content, owing to the presence of non-protein nitrogenous components, such as chlorophyll and inorganic nitrogen (46, 47).

Comparative studies have examined the protein content of algae using different methods (46, 47). These studies suggested that a more accurate nitrogen-to-protein conversion rate for algae is 5 (47, 48), or potentially closer to 4, depending on the specific species (46). After adjusting for the conversion rate, the highest recorded protein content in red seaweed should be 30 to 35%, which is closer to the typical protein content of *Porphyra* spp., which ranges from 23.7 to 30% (49–51).

The protein content of green seaweeds is comparable to that of red seaweeds, and brown seaweeds have a lower protein percentage of dry weight than these two, regardless of the protein determination technique (42, 48). According to a meta-analysis, the 5th to 95th percentile range for green seaweeds protein content is 4.6 to 32.2% dry weight, the range for red seaweeds is 2.0 to 28.7% dry weight, and the range for brown seaweeds is 3.3 to 15.9% dry weight (48). Nonetheless, it is crucial to acknowledge that variations in their concentrations have been observed, which can be attributable to factors like species, seasonal changes, and environmental conditions of growth (42, 52, 53).

As previously stated, microalgal species are known for their high protein content. Notably, species such as *Chlorella* spp., *Spirulina*, and *Dunaliella salina* (*D. salina*) have been extensively utilized, reporting a protein content of 38 to 70% dry weight (54–58). In contrast to seaweeds, protein content in cultivated microalgae remains consistent within standardized systems, although differences are observed within the same species based on the selected cultivation methodology (35, 59, 60).

The protein content of algae exceeds that of eggs (13%) and certain plant-based grains such as oats (13.5%) and wheat (15%), while being comparable to that of soy (38.6%) and chicken breast (31%) (61). Notably, it is important to acknowledge that algae protein encompasses the entire spectrum of EAA, which will be elaborated in Section 3.1.2.

#### 3.1.2. Quality of algal protein: amino acids composition

The primary determinants of a protein’s nutritional quality are its amino acid composition, their relative quantities, and their bioavailability, which reflects the body’s ability to absorb and utilize them (62, 63). Among the 20 distinct proteinogenic amino acids present in the human body, nine are classified as essential amino acids (EAAs) due to the body’s inability to synthesize them in quantities that adequately fulfill physiological requirements, making them necessary to be acquired through dietary sources (64). These are tryptophan, histidine, isoleucine, leucine, lysine, methionine, phenylalanine, threonine, and valine.

**Figure 2:**
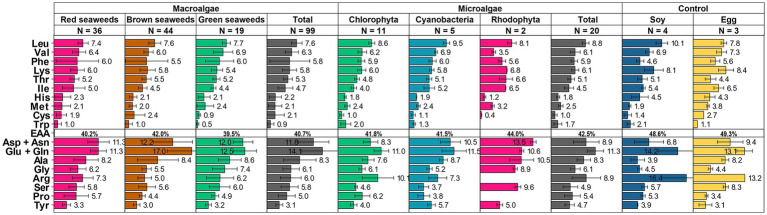
Amino acid compositions (expressed in grams per 100 grams of protein) of various macro- and microalgae were consolidated from scientific literature. The error bar represents the standard deviation (S.D.). N represents number of datapoints. Please refer to Supplementary Table S1 for the unprocessed data and corresponding scientific literature references.

On average, the composition of EAAs in algae meets the Food and Agriculture Organization (FAO) guidelines (65), and also closely resembles that of conventional protein sources, such as soybeans and eggs. Amino acid content of commercially available microalgae, such as *Chlorella*, Spirulina, *Nannochloropsis* spp., *Tetraselmis* spp., and macroalgae, dulse and *Porphyra* spp. are superior to that of the FAO guidelines (Supplementary Table S1), this is in contrast to many other plant-based proteins, which often fall short of meeting the recommended daily intake (66). On average, algae possess an EAA composition of 40 to 44%, which is marginally lower than soy at 48.6% and eggs at 49.3% (Figure 2).

A closer breakdown of amino acid composition is depicted in Figure 2. While amino acid profiles differ across algae species, the branched-chain amino acids (BCAAs)–namely leucine, isoleucine, and valine–demonstrate comparable levels to soy and eggs, making them a promising protein source for muscle health promotion.

However, algae are relatively deficient in sulfur-containing amino acids, such as cysteine and methionine. It is noteworthy that FAO/WHO/UNU recommends a daily intake of 13–15 mg/kg/day for these amino acids (67, 68), which is achievable with approximately 20 g of algal protein daily for an average weight adult. Algae also outperform soy in cysteine content, but not eggs. On the other hand, algae contain lower amounts of other EAAs like lysine and histidine compared to eggs and soy.

Conversely, algae are rich in aspartic acid and glutamic acid, especially in seaweed (Figure 2). High amounts of glutamic acid, typically ranging from 20 to 33% in certain genera like *Sargassum* and *Laminaria* (Supplementary Table S1), contribute to the distinctive ‘umami’ taste in seaweed, which can be further intensified when combined with 5′-ribonucleotides and alanine. Algae also have a higher content of alanine and glycine compared to soy and egg, which is important for physiological functions such as gluconeogenesis and as neurotransmitters. In addition, algae possess moderate levels of phenylalanine, tyrosine, and threonine, which are generally recognized as the limiting amino acids that restrict the nutritional value of plant proteins.

#### 3.1.3. Algae as a viable alternative protein source: safety and availability

The effects of consuming seaweed as an alternative protein source are still largely unknown in humans, particularly in terms of protein bioavailability and cellular utilization. Nevertheless, the utilization of macroalgae or seaweed biomass as alternative feed resources for livestock has gained increasing interest in recent years, yielding valuable preliminary information for humans. A meta-analysis of six animal studies involving broiler chickens revealed that dietary seaweed enhanced body weight gain and feed conversion ratio without affecting intake (69). The types of seaweed used varied, as did the inclusion dose (2 to 30 g/kg) and intervention duration (21 to 42 days). Notably, one of the initial investigations that introduced seaweed as a dietary component for chickens demonstrated that *Ulva* seaweed feeding resulted in reduced growth when included as 10% of the total diet, owing to its substantial unmetabolizable polysaccharide content (70). At a lower incorporation, a 3% *Ulva* diet increased the methionine composition in the feeds, which contributed to the increased muscle yield and serum quality. However, no significant effects were observed on feed intake or growth (71, 72). For ducks however, the inclusion of 12 to 15% red seaweeds *Polysiphonia* improved carcass muscle quality (73). In swine, the inclusion of seaweed resulted in improved immune function responses (74), and at a 1% inclusion level of *Ascophyllum*, muscle quality and gut health were improved (75, 76). *In vitro* digestibility of seaweeds ranged from 77 to 82% (77).

In terms of microalgae, the rigid cell wall limits protein digestibility and bio-accessibility in poultry and swine (78). According to Spínola et al. (78), protein digestibility of Spirulina (*A. platensis*) ranges from 66.1 to 68.7% in poultry (at 1% microalgae incorporation) and from 75.4 to 80.6% in swine (at 10% microalgae incorporation). However, when these cell walls are mechanically or enzymatically broken down through methods like bead milling or enzymatic hydrolysis, the digestibility significantly improves, as shown by *in vitro* digestion. For example, digestibility in *Nannochloropsis* spp. increased from 54 to 79% (79) and from 62 to 78% (80), as well as in *Chlorella* and *A. platensis*, it increased from 35 and 50% to almost 70 and 97%, respectively (81).

The highest administration of algal biomass is reported in a 4-week human trial examining the impact of administering 48 g of brown seaweeds (wakame) daily in the form of tablets (82). There were no reported adverse effects but instead, several positive effects were observed (82), which will be further discussed in Section 4. Due to the limited availability of algal protein on the market, there have been no clinical studies evaluating its consumption effects. It must be noted that while algae are generally regarded as safe for consumption, there have been documented instances of allergic reactions in the Allergome database (allergome.org) for species including *A. maxima*, *A. platensis*, *Chlorella* (species not specified), and *Laminaria*. Nevertheless, the allergenicity of algae is yet to be biochemically characterized and studied (54).

Although studies on algae’s efficacy as a protein source for humans are limited, collective findings of livestock studies suggest that it could be beneficial for weight gain, cellular utilization, and gut health maintenance. A human trial involving 48 g high doses of brown seaweeds biomass had no adverse reactions reported. Also, even though microalgae may have limited protein availability due to their rigid cell walls, this can be improved by pre-treatments, particularly beads milling. However, more research is needed to confirm algae’s safety and efficacy as a protein source for human consumption.

#### 3.1.4. Beyond protein: algae’s co-nutrients

While extracting pure protein from algae is challenging, algae’s rich co-nutrients such as carbohydrates, pigments, and lipids offer various health benefits beyond protein content (22, 35). These have been discussed elsewhere (83–92), and are not the focus of this study.

Regarding macronutrients, algae are rich in carbohydrate content due to their photosynthetic nature. Particularly in macroalgae, these saccharides appear as indigestible fibers, which act as prebiotics to promote peristalsis, enhance satiety, and slow down gastric emptying (22). Notably, saccharides in macroalgae such as *Neoporphyra haitanensis* (Chinese zicai), *A. nodosum*, *F. vesiculosus* and *S. japonica* are reported to maintain a healthy gut microbiome and modulate glycemic responses to starch intake (22, 93). Besides saccharides, lipids serve as energy stores as well as structural components in algae. Macroalgae typically contain less than 5% lipids, while some microalgae species contain 20 to 50% lipids (94, 95). The long-chain polyunsaturated fatty acids (PUFAs) found in algae hold particularly promising applications in nutraceuticals and pharmaceuticals as supplements, but their bioaccessibility might be limited by algal cell walls (96).

As for micronutrients, algae are considered superfoods as they contain a variety of vitamins including provitamin A, vitamins B1, B2, B6, B12, C, and E, as well as folic acid, niacin, and pantothenic acid. However, the bioavailability of algal vitamin B12 is questionable (97, 98). For example, dried laver/nori and *Chlorella* contain genuine vitamin B12 (97, 99, 100), whereas Spirulina contains mostly pseudo-B12, which is inactive in humans (101–104). Although pseudo-B12 is not harmful (105), it should be noted that other Spirulina components may interfere with vitamin B12 absorption (106), rendering it a less reliable B12 supplement. In addition, algae has the unique ability to form micelles (107, 108) and facilitate mineral uptake and absorption, as confirmed by *in vitro* digestion models (109, 110).

Bioactive compounds, such as carotenoids and polyphenols, are another important co-nutrient in microalgae and seaweeds. Notably, microalgae are a cost-effective source of a broad spectrum of carotenoids (111–113), including astaxanthin and lesser-known pigments like fucoxanthin and zeaxanthin (114, 115). Additionally, these organisms contain polyphenols such as phenolic acids, flavonoids, and lignans, which are potent antioxidants and anti-inflammatory agents (108–113, 116, 117) contributing to cellular protection from oxidative stress (23, 116, 118–123) and cardioprotective properties (124).

In summary, while the extraction of pure protein from algae may present challenges, algae offer a comprehensive array of macronutrients such as saccharides and lipids, and micronutrients such as vitamins, minerals, carotenoids, and polyphenols, which possess beneficial functions — from gut health enhancers to potential pharmaceutical resources. These highlight algae’s untapped potential in various health-related applications in metabolic effects, which will be discussed in Section 4.

## 4. Health effects of algae demonstrated in clinical trials

While *in vitro* and animal studies offer crucial insights into the prospective health benefits of algae — a topic thoroughly examined elsewhere (125) — clinical studies centered on the potential health benefits of incorporating algae into dietary routines present advantageous insights for nutritional science. To date, research has largely focused on examining the health effects of whole algal biomass while the health benefits of isolates, such as protein isolates, are unknown. Section 4 provides an overview of the health effects of algae demonstrated in human clinical trials with regards to glucose metabolism and satiety, lipid metabolism, and other health-promoting properties.

### 4.1. Effects of algae on glucose metabolism and satiety

Eleven studies that investigated effects of algae consumption on postprandial glucose homeostasis and satiety were reviewed (Table 1). Four were postprandial studies, two were short-term randomized controlled trials (RCTs), and five were longer-term RCTs. Findings showed disparities, with some studies demonstrating beneficial effects on glycaemia and satiety, while others found no significant impact.

**Table 1:** Summary of human trials on effects of algae consumption on glucose homeostasis and satiety.

Study population	Study design	Algae	Method of administration	Intervention	Duration	Study outcomes (Glucose and Satiety)	References
Healthy volunteers, 19–56 years, M/F, *n* = 38	Double-blinded, randomized 3-way crossover trial	*F. vesiculosus* (brown seaweed)	Capsules	Intervention 1: 500 mg *F. vesiculosus* Intervention 2: 2000 mg *F. vesiculosus* Control: 2000 mg cellulose placebo Study meal of 50 g available carbohydrate (white bread)	Acute postprandial study (2 h post-meal)	No effects on glucose or insulin. Asian participants had consistently ↑ insulin responses.	Murray et al. (126)
Healthy volunteers, 19–59 years, M/F, n = 23	Double-blinded, randomized 2-way crossover trial	*A. nodosum* and *F. vesiculosus* (brown seaweed)	Capsules	Intervention: 500 mg algae blend 1-week washout Control: 500 mg placebo Study meal of 50 g available carbohydrate (white bread)	Acute postprandial study (3 h post-meal)	↓ insulin and ↑ insulin sensitivity. No differences in glucose.	Paradis et al. (127)
Healthy volunteers, 20–50 years, M/F, *n* = 20	Blinded, randomized 3-way crossover trial	*L. digitata* (LD) and *U. pinnatifida* (UP) (brown seaweed)	Whole seaweed salads	5,000 mg of LD or UP or an energy-adjusted control meal containing pea protein.	Acute postprandial study (3 h post-meal)	↓ glucose, insulin, and C-peptide following LD and UP intake in participants <63 kg, after adjustment for body weight. LD and UP ↓ hunger and ↑ satiety but did not affect subsequent food intake.	Zaharudin et al. (128)
Overweight, otherwise healthy volunteers, 18–65 years, M, *n* = 12	Single-blinded, 2-way crossover trial	*A. nodosum* (brown seaweed)	Algae-enriched bread	Intervention: 100 g toasted bread enriched with 4% *A. nodosum* Control: 100 g toasted standard whole meal bread (0% *A. nodosum*) Both meals were consumed with scrambled eggs	Acute postprandial study (4 h post-meal)	↓ subsequent food intake. No changes in glucose.	Hall et al. (129)
Healthy volunteers, 18–45 years, M/F, *n* = 28	Double-blinded, prospective, randomized, 2-way crossover, pilot study	*A. nodosum* (brown seaweed)	Capsules	Intervention: Capsules containing 1,000 mg *Garcinia cambogia* (*G. cambogia*), 400 mg *A. nodosum* and 40 mg L-carnitine in total for 1 week 1-week washout Control: Placebo capsules similar in weight, color, and size to intervention for 1 week	2 weeks (excluding 1-week washout)	↓ hunger and satiety; ↑ fullness and preference for sweet foods.	Mayer et al. (130)
Type 2 diabetes mellitus patients, 40–70 years, M/F, *n* = 20	Randomized controlled trial	Sea mustard (also known as wakame or *U. pinnatifida*, a brown seaweed) and sea tangle	Pills	Intervention: 48 g algae/day Control: No supplementation	4 weeks	↓ fasting glucose and 2-h postprandial glucose. No changes in HbA1c.	Kim et al. (82)
Participants with at least 1 symptom of the metabolic syndrome, men (47.4 ± 9.9 years), women (45.6 ± 12.2 years), M/F, *n* = 27	Double-blinded, 2-way crossover study	*U. pinnatifida* (brown seaweed)	Capsules	Group 1: 1 month of maltodextrose placebo followed by 1 month of 4,000 mg/day algae Group 2: 1 month of 4,000 mg/day algae followed by 1 month of 6,000 mg/day algae	8 weeks	No changes in glucose, insulin, or HOMA-IR.	Teas et al. (131)
NAFLD patients, 20–50 years, M/F, *n* = 55	Double-blinded, randomized, controlled clinical trial	*C. vulgaris* (green microalgae)	Tablets	Intervention: 1,200 mg/day *C. vulgaris* + 400 mg/day Vitamin E Control: Placebo tablets +400 mg/day Vitamin E	8 weeks	↓ glucose. No significant changes in insulin and HOMA-IR.	Ebrahimi-Mameghani et al. (132, 133)
Hypercholesterolemia patients, 20–60 years, M/F, *n* = 103	Double-blinded, randomized, controlled and parallel-group comparison trial	*P. palmata* (red seaweed)	Capsules	Intervention: 2000 mg/day *P. palmata* Control: Placebo capsules	8 weeks	No differences in glucose, glycated albumin, insulin, or HOMA-IR.	Takase et al. (134)
Type 2 diabetes mellitus patients, 20–65 years, M/F, *n* = 84	Double-blinded, randomized controlled trial	*C. vulgaris* (green microalgae)	Capsules	Intervention: 1,500 mg/day *C. vulgaris* Control: 1,500 mg/day placebo	8 weeks	No changes in glucose, insulin, HbA1c or HOMA-IR.	Hosseini et al. (135)
Intervention: Non-insulin dependent diabetes mellitus (NIDDM) patients, mean age: 47.8 years, M/F, *n* = 15 Control: Healthy volunteers, Mean age: 53.4 years, M/F, *n* = 7	Controlled clinical trial	Spirulina (blue-green algae; species not specified)	Tablets	Intervention: 2000 mg/day Spirulina Control: Not supplemented with Spirulina	8 weeks	↓ glucose	Mani et al. (136)
At-risk group: Volunteers with high risk of diabetes or hyperlipidemia, > 20 years, M, *n* = 17 Healthy group: Healthy volunteers, > 20 years, M, *n* = 16	Clinical trial	*C. pyrenoidosa* (green microalgae)	Tablets	Both groups took 8,000 mg/day of Sun Chlorella A	12 weeks (excluding 4-weeks follow-up)	↓ glucose; no changes in insulin.	Mizoguchi et al. (137)
NAFLD patients, 35–70 years, M/F, *n* = 54	Randomized open-label clinical trial	*C. vulgaris* (green microalgae)	Tablets	Intervention: 1,200 mg/day *C. vulgaris* + 750 mg/day metformin +200 mg/day Vitamin E Control: 1,250 mg/day metformin +200 mg/day Vitamin E	12 weeks	↓ HbA1c and HOMA-IR in intervention group. ↓ glucose in control group.	Panahi et al. (138)
Pre-diabetic volunteers, 20–60 years, M/F, *n* = 80	Double-blinded, randomized, clinical trial	*Ecklonia cava* (brown seaweed; referred hereafter as *E. cava*)	Tablets	Intervention: 1,500 mg/day dieckol-rich extract from *E. cava* Control: 1,500 mg/day placebo	12 weeks	↓ glucose	Lee et al. (139)
Overweight or obese prediabetic volunteers, 18–70 years, M/F, *n* = 56	Double-blinded, randomized, parallel clinical trial	*A. nodosum* and *F. vesiculosus* (brown seaweed)	Capsules	Intervention: 500 mg/day brown algae extract Control: 500 mg/day placebo Both groups also received individualized nutritional advice for moderate weight loss	12 weeks	No effect on glucose; ↓ C-peptide at 120 min-OGTT.	Vodouhè et al. (140)

#### 4.1.1. Postprandial studies

In acute postprandial crossover studies involving healthy participants, hypoglycemic effects of algae were more evident in participants of lower body weight and in studies lasting at least 3 h after consuming a carbohydrate-based meal (Table 1). For instance, Murray et al. found no discernible hypoglycemic effect within the first 2 h of consuming up to 2000 mg brown algae (126), whereas another study found evident hypoglycemic effects within 3 h following ingestion of 250 mg brown algae extract (127).

Satiating effects of algae were demonstrated, although potentially influenced by the macronutrient composition of the study meal. A longer, 4-h postprandial study found no changes in glycaemia but distinct changes in satiety (129). This may be due to the relatively small sample size (n = 12) and that the study meal served scrambled eggs with the algae-enriched bread, increasing the protein and fat content of the meal as compared to other studies that served algae with white bread (126, 127). Another 3-h postprandial study saw no hypoglycemic effects until body weight was factored into the statistical analysis (128). Consuming the algae also had satiating effects though it did not affect subsequent food intake.

#### 4.1.2. Short-term randomized clinical trials

Two studies ranging from 2 to 4 weeks found algae to increase satiety, reduce fasting glucose and decrease 2-h postprandial glucose (Table 1). Both studies utilized brown algae, but varied in daily dosages and the health status of participants — ranging from 400 mg/day in healthy participants (130) to 48 g/day in diabetic patients (82). These variations, combined with the different metrics employed across studies, limit the comparability of these studies.

#### 4.1.3. Longer-term randomized clinical trials

RCTs ranging from 8 to 12 weeks in duration found inconsistent results regarding hypoglycemic effects following algae intake (Table 1). However, reductions in glucose, HbA1c and HOMA-IR appeared more consistently in 12-week studies. The heterogeneity in these longer-term trials’ results could be attributable to suboptimal dosages of algae intake. Specifically, in the study conducted by Vodouhè et al. (140), the absence of effect on blood glucose could be due to the lower algae dose, compared to other studies of similar duration.

The study conducted by Mizoguchi et al. (137) was conducted in the absence of a proper control. It remains unclear whether the observed alterations in glycemic levels can be attributed to the consumption of *Chlorella* tablets or if they were influenced by the elevated susceptibility to disease. It also should be noted that the majority of these longer-term trials were conducted in individuals with or at high risk of chronic diseases. Hence, disease state and physiological differences may account for some inconsistencies in glycemic effects. Furthermore, the restricted scope of certain trials, such as those conducted among Mestizos in South America (131) or diabetes patients in clinical settings (136), may limit their generalizability.

Anti-diabetic properties of brown algae may be through polysaccharides, phlorotannins and polyphenolics that inhibit carbohydrate-digesting enzymes α-amylase and α-glucosidase and decrease hepatic gluconeogenesis. This has been demonstrated *in vitro* and in animal studies (141–143). *Chlorella*’s glucose-lowering properties may be attributed to decreasing non-esterified fatty acid levels, hence improving glucose uptake and utilization, supported by results in animal studies (144, 145). Antioxidants including lutein, α-tocopherol, ascorbic acid, and α- and β-carotene, may also improve insulin sensitivity and hepatoprotection (146–148).

Bromophenols found in red algae exhibited anti-diabetic activities in animal models by inhibiting α-glucosidase and PTP1B (149). Despite these promising findings, Takase et al. (134) found no changes in glucose homeostasis with 2000 mg/day red algae after 8 weeks although improvements in serum triglyceride (TG) after this first mention of triglyceride were seen in women. It could be that brown algae have more potent hypoglycemic effects.

However, anti-diabetic mechanisms of algae in humans are yet to be determined since findings have been inconsistent, such as consuming 500 mg algae extract resulting in hypoinsulinemic responses (127) but not after 2000 mg of algae (126). Moreover, studies have shown trends such as improvements in postprandial insulin but not in glucose levels (127).

Different algae dosages and compositions of the delivery medium could also explain variabilities in glycemic responses (126, 127), as capsules used in Paradis et al. contained algae extracts which were free of alginates and partially demineralized. In the study conducted by Zaharudin et al. (128), the relatively unprocessed whole seaweed salads used could explain the lack of postprandial glycemic responses before weight adjustment.

Taken together, there are demonstrated hypoglycemic and satiating effects of algae. However, results are inconsistent and further research is needed to establish more definitive conclusions and determine the optimal dosage and duration of algae consumption for glycemic control and effects on satiety.

### 4.2. Effects of algae on lipid metabolism

#### 4.2.1. Short-term randomized clinical trials

Algae consumption has been associated with improvements in lipid profile across multiple studies (134, 136, 137, 150–154). In RCTs ranging 4 to 8 weeks, various forms of algae, including microalgae Spirulina and *C. vulgaris*, and seaweeds *E. cava*, *Ulva*, and sea mustard (*U. pinnatifida*), have demonstrated beneficial impacts on lipid profiles. Key outcomes across these studies highlight reductions in TG, total cholesterol, LDL-C, and VLDL-C.

#### 4.2.2. Longer-term randomized clinical trials

All 12-week RCTs saw decreases in total cholesterol and LDL-cholesterol when administered with 2 to 8 g of microalgae (137, 150) or 400 mg of brown seaweed extract (153). An exception was Murray et al. (155) which provided 2000 mg brown seaweed extract but was underpowered to detect changes in lipid parameters. These findings span diverse populations, including healthy volunteers and at-risk populations with heart disease, diabetes, and those with hypercholesterolemia, reinforcing the potential broad-spectrum benefits of algae supplementation. These beneficial effects vary with the species and dosage of algae and the individual’s health status.

Notably, one study by Roach et al. (154) conducted a study investigating different dosages, revealing that lower doses (2 g) of sulfated xylorhamnoglucuronan-rich seaweed extract (*Ulva* sp. 84) yielded better outcomes than higher doses (4 g) in overweight volunteers. A subsequent study was then carried out with increased power to validate the reduction in non-HDL cholesterol seen, but results were not corroborated (154). The authors posited that it was potentially due to the baseline metabolic health of the study participants being less compromised (154). In addition, no washout period between the intervention arms might be another reason contributing to the unclear results of the trials. These findings underscore the need for personalized and precise dosing strategies in optimizing the lipid-lowering benefits of algae.

Specifically, the beneficial effect of Spirulina on lipid metabolism can be attributed to its high content of γ-linolenic acid, protein, and fiber. γ-linolenic acid inhibits the buildup of fat and cholesterol in the body. The protein and fiber composition of Spirulina lowers the production of VLDL and TG, as well as promotes the clearance of VLDL in the periphery, leading to declines in TG and VLDL after Spirulina supplementation. This in turn decreases the amount of LDL since the majority of these are generated from VLDL (136). It has been proposed that the positive effect of *E. cava* on blood lipid profiles is due to its polyphenol or phlorotannin content, although the mechanisms are unclear (153). The beneficial effect of SXRG84 on lipid levels can be attributed to the generation of short-chain fatty acid propionates by *Akkermansia* and *Bacteroides*, both of which increased in Study 1 (154). Further studies are necessary to explore and confirm the mechanisms behind the anti-dyslipidemic effects (Table 2).

**Table 2:** Summary of human trials on effects of algae consumption on lipid metabolism.

Study population	Study design	Algae	Method of administration	Intervention	Duration	Study outcomes (Blood Lipids)	References
Intervention: Non-insulin dependent diabetes mellitus (NIDDM) patients, mean age: 47.8 years, M/F, *n* = 15 Control: Healthy volunteers, Mean age: 53.4 years, M/F, *n* = 7	Controlled clinical trial	Spirulina (blue-green algae; species not specified)	Tablets	Intervention: 2000 mg/day Spirulina Control: Not supplemented with Spirulina	8 weeks	↓ TG, TC, LDL-C and VLDL-C.	Mani et al. (136)
Patients with ischemic heart disease and hypercholesterolemia, 40–60 years, M/F, *n* = 30	Controlled clinical trial	*Arthrospira fusiformis* (blue-green algae)	Tablets	Intervention 1: 2000 mg/day *A. fusiformis* Intervention 2: 4,000 mg/day *A. fusiformis* Control: Not supplemented with *A. fusiformis*	12 weeks	↓ TC, LDL-C, TG, VLDL-C; ↑ HDL-C.	Ramamoorthy and Premakumari (150)
Healthy volunteers, > 20 years, M/F, *n* = 29	Double-blinded, randomized, placebo-controlled study	*C. vulgaris* (green microalgae)	Tablets	Intervention: 5,000 mg/day *C. vulgaris* Control: 5,000 mg/day placebo (lactose) Consumption of 3 eggs/day to provide 510 mg cholesterol daily	4 weeks	Suppressed elevation in TC and LDL-C.	Kim et al. (151)
At-risk group: Volunteers with high risk of diabetes or hyperlipidemia, > 20 years, M, *n* = 17 Healthy group: Healthy volunteers, > 20 years, M, *n* = 16	Clinical trial	*C. pyrenoidosa* (green microalgae)	Tablets	Both groups took 8,000 mg/day of Sun Chlorella A	12 weeks (excluding 4-weeks follow-up)	↓ TC, LDL-C and HDL-C in volunteers with high-risk factors for lifestyle-related diseases	Mizoguchi et al. (137)
Healthy volunteers with mild hypercholesterolemia, mean age: 49.8 years (Intervention), 49.8 years (Placebo), M/F, *n* = 63	Randomized double-blind, placebo-controlled trial	*E. cava* (brown seaweed)	Tablets	Intervention: 400 mg *E. cava* extract/day Control: placebo tablet containing no *E. cava* extract	12 weeks	↓ TC and LDL-C.	Choi et al. (153)
Type 2 diabetes mellitus patients, 40–70 years, M/F, *n* = 20	Randomized controlled trial	Sea mustard (also known as wakame or *U. pinnatifida*, a brown seaweed) and sea tangle	Pills	Intervention: 48 g algae/day Control: No supplementation	4 weeks	↓ TG and ↑ HDL-C.	Kim et al. (82)
Study 1: Overweight or obese volunteers (BMI 25-40 kg/m^2^), ≥ 18 years, M/F, *n* = 64 Study 2 Overweight volunteers (BMI 25-29 kg/m^2^), ≥ 18 years, M/F, *n* = 64	Study 1: Double-blinded, randomized, controlled trial Study 2: Double-blinded, randomized, 2-way crossover trial	*Ulva* sp. 84 (green seaweed)	Capsules	Study 1: Intervention 1: 2000 mg SXRG84/day Intervention 2: 4,000 mg SXRG84/day Study 2: Intervention 1: 2000 mg SXRG84/day for 6 weeks Control: Placebo for 6 weeks	Study 1: 6 weeks Study 2: 12 weeks (no washout period)	Study 1: ↓ non-HDL-C in overweight volunteers consuming 2000 mg SXRG84/day Study 2: No differences in lipid markers	Roach et al. (154)
Overweight and obese adults with elevated LDL-C (> 2.0 mmol/L), 18–65 years, M/F, *n* = 34	Double-blinded, randomized, controlled trial	*F. vesiculosus* (brown seaweed)	Capsules	Intervention: 2000 mg *F. vesiculosus* Control: 2000 mg rice flour placebo	12 weeks	No changes in TC, TG, HDL-C, LDL-C or TC:HDL ratio.	Murray et al. (155)

### 4.3. Other health-promoting properties

Consuming algae has overall beneficial effects on inflammation and gut health in humans, although the available evidence varies in study design and algae dose administered. Nine studies were reviewed, with three of them investigating gut health (154, 156, 157).

#### 4.3.1. Short-term randomized clinical trials

Four short-term studies showed improved anti-inflammatory markers and gut health after algae ingestion (Table 3). This was apart from one study that found algae-enriched bread to increase (as opposed to decrease) serum C-reactive protein (CRP) levels (158). As CRP levels remained within the normal clinical range, the authors stated that this was unlikely to impact health. Studies ranged from 2 to 4 weeks of intervention and were in generally healthy participants apart from one small study with Type 2 diabetes patients (82).

**Table 3:** Summary of human trials on effects of algae consumption on other health-promoting properties.

Study population	Study design	Algae	Method of administration	Intervention	Duration	Study outcomes (Other Health-Promoting Properties)	References
Volunteers with infrequent bowel movements (3-5x weekly), 20–65 years, M/F, *n* = 38	Double-blinded, randomized clinical trial	*M. nitidum* (green seaweed)	Capsules	Intervention: 100 mg of RS (Rhamnox), a sulfated polysaccharide from *M. nitidum* Control: Placebo	2 weeks	↑ excretion frequency, ↑ excretion days. In intervention group: ↑ excretion frequency in those with ↑ BMI, weight, or gut microbiota diversity. ↑ excretion days in those with ↑ BMI.	Shimada et al. (156)
Healthy volunteers, 18–75 years, M/F, *n* = 40	Randomized clinical trial	*L. japonica* (brown seaweed)	*L. japonica* – tablet Duolac7S – capsule Duolac7S is a probiotic mixture of lactic acid bacteria	Intervention: *L. japonica* (1,250 mg/day) with Duolac7S Control: *L. japonica* (1,250 mg/day) with corn starch placebo	4 weeks (excluding 2-week follow-up)	↑ in 4 intestinal microbiota species. No changes in gastrointestinal symptoms or bowel functions.	Ko et al. (157)
Healthy volunteers, 18–65 years, M/F, *n* = 35	Double-blinded, randomized clinical trial	*P. palmata* (red seaweed)	Algae-enriched bread	Intervention: 5,000 mg/day *P. palmata* incorporated into bread Control: Bread of the same composition as intervention bread but without *P. palmata.*	4 weeks	↑ C-reactive protein	Allsopp et al. (158)
Type 2 diabetes mellitus patients, 40–70 years, M/F, *n* = 20	Randomized controlled trial	Sea mustard (also known as wakame or *U. pinnatifida*, a brown seaweed) and sea tangle	Pills	Intervention: 48 g algae/day Control: No supplementation	4-week intervention	↓ TBARS; ↑ catalase and GSH-Px.	Kim et al. (82)
Postmenopausal volunteers, 47–54 years, F, *n* = 21	Clinical trial	Klamath algae (*A. flos-aquae*) (blue-green algae)	Tablets	1,600 mg algae extract/day	8 weeks	↓ lipid peroxidation; ↑ carotenoids, tocopherols, and retinol.	Scoglio et al. (159)
NAFLD patients, 20–50 years, M/F, *n* = 55	Double-blinded, randomized, controlled clinical trial	*C. vulgaris* (green microalgae)	Tablets	Intervention: 1,200 mg/day *C. vulgaris* + 400 mg/day Vitamin E Control: Placebo tablets +400 mg/day Vitamin E	8 weeks	↓ hs-CRP	Ebrahimi-Mameghani et al. (132)
Overweight or obese prediabetic volunteers, 18–70 years, M/F, *n* = 56	Double-blinded, randomized, parallel clinical trial	*A. nodosum* and *F. vesiculosus* (brown seaweed)	Capsules	Intervention: 500 mg/day brown algae extract Control: 500 mg/day placebo Both groups also received individualized nutritional advice for moderate weight loss	12 weeks	Inhibited ↑ in IL-6. No changes in hs-CRP and F2-isoprostane.	Vodouhè et al. (140)
Study 1: Overweight or obese volunteers (BMI 25-40 kg/m^2^), ≥ 18 years, M/F, *n* = 64 Study 2 Overweight volunteers (BMI 25-29 kg/m^2^), ≥ 18 years, M/F, *n* = 64	Study 1: Double-blinded, randomized, controlled trial Study 2: Double-blinded, randomized, 2-way crossover trial	*Ulva* sp. 84 (green seaweed)	Capsules	Study 1: Intervention 1: 2000 mg/day SXRG84 Intervention 2: 4,000 mg/day SXRG84 Study 2: Intervention 1: 2000 mg/day SXRG84 for 6 weeks Control: Placebo for 6 weeks	Study 1: 6 weeks Study 2: 12 weeks (no washout period)	Study 1: ↓ CRP with 4,000 mg/day dose in overweight participants. Gut flora shifts in pooled 2000 and 4,000 mg/day SXRG84 compared to placebo. No changes in F2-isoprostanes. Study 2: ↓ pro- inflammatory and anti-inflammatory cytokines. No differences in gut microbiota.	Roach et al. (154)
Overweight and obese volunteers BMI ≥ 25 kg/m^2^, 30–65 years, M/F, *n* = 78	Double-blinded, randomized, 2-way crossover trial	*A. nodosum* (brown seaweed)	Capsules	Intervention: 400 mg/day capsule containing 100 mg algae (poly)phenol and 300 mg maltodextrin for 8 weeks 8-week washout Control: 400 mg/day maltodextrin placebo capsule for 8 weeks	16 weeks (excluding 8-week washout)	↓ basal DNA damage in obese participants and in men; ↓ peroxide in women. No other changes in CRP, antioxidant status or inflammatory cytokines.	Baldrick et al. (160)

#### 4.3.2. Longer-term randomized clinical trials

Longer term trials consistently demonstrated anti-inflammatory properties of algae, typically through reduced lipid peroxidation, lowered CRP, favorable changes in cytokines, increased antioxidant levels and reduced DNA damage (Table 3). Studies ranged from 6 to 16 weeks and were generally larger studies apart from one small trial (*n* = 21) (159). Roach et al. (154) found significant gut flora shifts after 6 weeks, although results were not replicated in a subsequent crossover trial. This was likely due to a lower algae dose (2000 mg/day) whereas the previous study used double the dose and pooled the intervention groups to see these microbiota changes. The subsequent crossover trial had no washout period between the two study arms however, increasing the likelihood of carry-over effects.

How algae reduce inflammation is unclear but *in chemico*, *in vitro* and *in vivo* animal studies suggest algae-derived bioactive compounds such as lipids have a role in mechanisms leading to downregulation of pro-inflammatory enzymes and mediators (89). The immunostimulatory activity observed by Allsopp and colleagues contrasts with previous *in vitro* and animal studies that have shown red algae to exert anti-inflammatory activity (161–164). Authors theorized that high temperatures in the baking process could have inactivated anti-inflammatory agents such as ω-3 PUFAs (158).

Beneficial effects of algae on gut health may be through their nutritional make up. The polysaccharide and fiber content of RS may explain its therapeutic effect on constipation, as well as causing functional alterations of cellular pathways (156). Authors noticed decreases in gut bacteria *Clostridiales* and increases in *Negativicutes* and *Acidaminococcales* which have been demonstrated to alleviate constipation (165, 166). In Roach et al. (154), gut flora changes were likely due to colonic bacteria responding positively to soluble dietary glycans, with some bacteria growing specifically on amine-polysaccharides provided during the study. Significant changes in intestinal microbiota however do not necessarily translate into improved gut health (157), although changes may be observed in studies of longer duration.

Studies varied greatly in algae dosages administered, from 100 mg to 48 g per day. Differing study durations and algae species may also explain why dose-dependent effects were not always observed. For example, 1,250 mg/day resulted in no gastrointestinal or bowel changes after 4 weeks (157) whereas constipation improved after 2 weeks of 100 mg/day of RS (156). Different algae species and a probiotic mixture given with the 1,250 mg/day of algae (157) may have led to distinct mechanisms explaining the differences in effects on the gut. A lower algae dose of 500 mg/day brown algae could explain why no changes in hs-CRP were seen after 12 weeks (though an improvement in IL-6 was seen) (140), when 1,200 mg/day *C. vulgaris* reduced hs-CRP after 8 weeks (132). Apart from Allsopp et al. (158), the reviewed studies investigating effects of algae on inflammation outcomes recruited diseased, postmenopausal, or overweight/obese patients who experience a basal level of inflammation which may not be present in healthy individuals. Hence factors such as disease state and physiological differences may explain some of the differences in observed anti-inflammatory changes in participants.

Several trials (135, 138, 140, 155, 158) measured blood pressure but found no difference between intervention and control groups. Teas et al. (131) observed reduced blood pressure though this was only in systolic blood pressure and in participants who consumed 6 g seaweed per day. However, significant reductions in systolic blood pressure were also seen in the placebo group but not in those consuming 4 g seaweed per day.

Algae has beneficial anti-inflammatory and prebiotic properties. However, studies with dose-dependent approaches and similar durations are needed to further define these effects and their underlying mechanisms. A recent meta-analysis of 31 trials found algae supplementation to significantly decrease HbA1c, HDL-C and TC and increase insulin, but not significantly change other glucose and lipid parameters (167). Results also suggested that algae supplementation could reduce 2-h post-meal glucose in Asians. Our findings support the decline in TC but observed discrepancies in the effects on other glycemic and lipid markers. These inconsistencies can potentially be explained by several factors, including the inclusion of different studies by Ding et al. in the meta-analysis, different algae species and dosages used, as well as dissimilar study populations. Moreover, several publications reviewed in the current work were published after December 2019 and therefore not included in the meta-analysis. However, Murray et al. (126) also observed ethnic-specific glucose metabolism responses in Asians versus non-Asians. Consistently elevated plasma insulin responses in Asian participants were found, irrespective of the algae dose (500 mg or 2000 mg) and even after a cellulose placebo, suggesting an increased risk of insulin resistance in this ethnic group compared with non-Asians. Hence, ethnicity may play a role in the effects of algae on postprandial glycaemia.

Taken altogether, clinical studies on algae consumption provide valuable insights into its potential health benefits and contribute to our understanding of the role of algae in promoting human well-being. From the clinical studies reviewed, algae intake has been shown to demonstrate hypoglycemic and satiating effects, improved lipid metabolism and beneficial effects on inflammation and gut health.

In spite of these encouraging results, additional research is required to determine the ideal dosage, long-term effects, processes, and potential treatments for increasing the bioavailability of nutrients from algae. A comparison of these metabolic effects utilizing the same algae in a dose-dependent manner with comparable study designs and durations is necessary due to the huge variances evident in prior research. Human clinical trials investigating similar effects for non-brown algae and algal extracts other than the total seaweed biomass, especially as protein extracts, are scarce and warrant further research efforts.

## 5. Incorporating algal proteins in future foods: challenges and opportunities

The food industry is witnessing an increase in innovative food products developed from algal biomass, marking the trend’s global progression (168). This interest stems from the nutritional characteristics that algae provide, as well as the previously noted health benefits (167–169).

In recent years, there has been a great deal of scientific interest in investigating the nutritional supplementation of algae in various cuisines. Due to the high operating costs associated with extracting and purifying algal proteins that maintain the nutritional and functional qualities of these proteins, the food industry focuses primarily on the utilization of algal whole biomass (19, 167, 168). Nonetheless, breakthroughs in the field of algal biomass incorporation in food production have revealed preliminary indicators of the modifications induced in the physiochemical features of final products and the resulting consequences, which will be elaborated below.

### 5.1. Algae-derived ingredients as flavor additives and seasonings

Incorporating algae-based ingredients in food additives and seasonings provides an opportunity to develop functional foods with added health benefits (22). Algae play a central role as ingredients in a wide range of dishes, providing unique flavors and a characteristic umami touch. Algae, in different forms, are extensively utilized in numerous recipes, particularly in health-conscious options. For example, algae can be added to citrus and fruity smoothies, salads, soups, and sushi recipes, enriching their nutritional value while imparting distinctive flavor profiles.

The edible kelp known as kombu, has gained significant popularity as food additives and flavor enhancers. Dr. Kikunae Ikeda, a prominent Japanese scientist, is credited with discovering the umami flavor from kombu dashi, a broth derived from kombu, in 1907. Kombu is a remarkable source of umami flavor, distinguished by its substantial content of all three key umami components: glutamate, inosinate, and guanylate. The notable attributes of kombu have resulted in its extensive utilization as a culinary enhancer across diverse global cuisines. Incorporating algae into homemade seasoning blends presents an intriguing opportunity to regulate sodium consumption, circumvent superfluous additives, and introduce the distinctive flavor characteristics inherent in algae.

### 5.2. Technofunctional properties of algal ingredients for food applications

Algae are significant in food technology due to their functional properties. Algal hydrocolloids like agar, carrageenan, xanthan, and guar gum are vital, serving as thickening, gelling, emulsifying, and stabilizing agents (170). Attributed to their ability to thrive in high stress living environments, algae produce an array of bioactive compounds, including antioxidants, polyphenols, and pigments.

In addition, algae, particularly microalgae, are abundant sources of various pigments, including chlorophyll, carotenoids, and phycobiliproteins (171). Due to the rising consumer demand for clean-label products and growing concerns about the potential health risks of synthetic colorants (172), algal pigments have become increasingly appealing to the food sector as natural alternatives (173, 174). Their application extends to various foods and beverages, ranging from chewing gum (175) to cookies (176).

Additionally, the natural encapsulation of algal pigments has developed into a revolutionary approach to protect pigments against deterioration and enhance their effectiveness in various food applications (177). Encapsulated pigments allow controlled and targeted release, preserving their stability and prolonging their functional properties in several dietary applications (177), such as antioxidant activities and other health-promoting effects (173, 177). Based on algal natural film-forming properties, algal-derived hydrocolloids have been developed into edible coatings and microcapsules for functional food products, thereby protecting bioactive ingredients from environmental damage, ensuring stability, and controlling release during consumption (178).

In addition to their effects on gut health, the dietary fibers present in algae, including polysaccharides, oligosaccharides, lignin, cellulose, and hemicellulose, have pivotal roles not only in the food industry but also in the cosmetic sector. These fibers contribute to the consistency of food products and act as stabilizing agents (22, 93, 179, 180).

### 5.3. Utility of algae in the production of meat analogs

Algal proteins also serve as a promising ingredient in meat analogs, not only for their physiochemical properties that serve as binders, fillers, and flavoring agents, but also for their amino acid profile that is promising in further bolstering nutritional value (181). Moreover, they provide a high-quality alternative for individuals allergic to soy or those adhering to vegan diets. Notable is the innovative utilization of algal ingredients to expand the selection of alternative plant-based meat products that are currently lacking on the market, particularly “pork” equivalents, such as dumplings and bacon, as well as seafood analogs (182, 183).

Algal biomass can be added in the production of meat analogs. Caporgno et al. demonstrated that mixing *Chlorella*-based microalgae with soy concentrates results in extrudates with a fibrous texture (184). Extrusion with up to 50% dried microalgal biomass affected fiber formation but could be mitigated by adjusting moisture levels. Microalgal-incorporated extrudates have a softer texture, due to the higher fat content in microalgae biomass resulting in lubrication effects while intact microalgae cells function as passive fillers —limiting access to intracellular proteins. Both features reduced texturing but increased tenderness compared to soy-based extrudates (184). Furthermore, the microalgae fortification enhanced the concentration of vitamins B and E in the extrudate, with over 95% preserved in the final product (184).

Similarly, incorporating Spirulina (*A. platensis)* biomass (10, 30% or 50%) in a texturized soy-based meat analog resulted in products with a black color and pronounced earthy flavor. However, 50% Spirulina (*A. platensis)* addition hindered the texture and led to reductions in the elasticity, fibrousness, and firmness of the extruded samples (183).

Interestingly, a red seaweed strain was patented for a strong bacon taste and later commercialized as a healthier vegan “bacon” product that is highly nutritious and sustainable (185, 186). For minced meat-based products, a commercially available, red-colored algal biomass known as Essential Red has been utilized. This biomass is derived from a specific microalgal strain with upregulated protoporphyrin IX and heme production pathways. Essential Red serves as the foundation for developing various algal-based animal-free “pork,” including vegan “meatballs,” pulled “pork” burgers, and dumplings with green-algal wrapping and red-algal filling (187).

Triton Algae Innovations, a company specializing in freshwater algae, exploits different strains of *Chlamydomonas reinhardtii* to produce algal-based biomass tailored to the needs of food companies it works with, including the Essential Red, Essential Green, and even colorless white algal biomass. The company selects strains with the desired biological traits, eliminating the need for genetic engineering, which is different from previous attempts in plant-based meat to mimic meat features (187, 188).

Besides plant-based proteins, algae’s utility in cultivated meat has been explored by Mewery, a food tech start-up venture that focuses on offering cultured pork products. Mewery has developed a novel prototype of cultivated meat composed of 75% pork cells and 25% microalgae cells. The incorporation of microalgae extracts in a hybrid culture medium provides the necessary conditions and nutrients for cell growth and division, allowing cells to develop into muscle and fat cells. Bioreactors have been built to scale up production and offer a diverse range of pork products, including meatballs, sausages, dumpling ground meat, and tenderloin (189, 190).

### 5.4. Algal protein fortification of carbohydrate-rich foods

#### 5.4.1. Pastas and noodles

Numerous studies have demonstrated that the incorporation of algae into pasta or noodles significantly enhances its nutritional value. Specifically, improvements in protein and amino acid profile (191, 192), lipid profile (192, 193), minerals (192, 194) and antioxidants (195) have been documented.

Incorporating seaweeds such as sea lettuce (*U. lactuca*), nori (*P. tenera*), and wakame (*U. pinnatifida*) into pasta, despite a low supplementation level of 3%, resulted in noticeable color changes, shorter cooking times, and texture modifications, including decreased hardness and increased adhesiveness and resilience. These additions also increased the protein content and soluble fiber of the pasta (194).

Studies investigating microalgae supplementation into pasta production include Spirulina (*A. platensis*), *D. salina* (196), *C. vulgaris* (191), *I. galbana* and *Diacronema vlkianum* (*D. vlkianum*) (193). Spirulina supplementation at concentrations between 7 and 12.5% yields pastas with darker, greener colors but higher consumer acceptance scores (197–199). Concentrations exceeding 15%, however, impact the sensory attributes of the pasta (199, 200).

Taken altogether, approximately 10% microalgae supplementation represents an optimal concentration for nutritional enhancement without hindering sensory acceptability. However, it is noteworthy that the algae species selection significantly influences the taste and hence consumer preference, as observed for a perceived seafood taste caused by inclusion of *I. galbana* and *D. vlkianum* despite no other changes in sensory perception (193).

The incorporation of microalgae into noodles, however, has shown lower acceptability. There have been commercial activities to add Spirulina into noodles (201) including instant noodles (202), with quantities ranging from 0.1 to 2%. It was reported that the noodles were blue-green in color with a mild seafood taste and favorable mouthfeel (201). However, incorporating higher quantities of microalgae (2 to 8% *A. platensis*), has been associated with decreased overall acceptability (192). Within the range of 2 to 4% Spirulina *(A. platensis)* supplementation, acceptability scores did not exhibit significant disparities. However, as the proportion of Spirulina *(A. platensis)* exceeded 4%, there was a proportional decline in the overall acceptability of the noodle products (192).

#### 5.4.2. Bakery products

##### 5.4.2.1. Bread

Various attempts have been made to enhance the nutritional value of bread by incorporating microalgae at different concentrations. These efforts have resulted in improved nutritional quality, particularly in terms of increased protein content, minerals, and ash content (203–208). Different effects were reported for the levels of macronutrients such as fat, crude fiber, and carbohydrates, and compounds such as carotenoids and luteins, depending on the strain and concentration used (205–208).

Algal biomass pigments, such as chlorophylls and carotenoids, have a significant impact on the color of the fortified bread (205–208). This might be perceived negatively by consumers due to the unexpected color change in a traditionally pale product like bread. All sensory evaluations showed that a lower percentage (1 to 2%) of microalgae-supplemented bread was preferred in terms of color, compared to more enriched bread (3 to 5%) (205, 206, 208).

In terms of taste profile, there is no significant difference between bread with 1 and 4% Spirulina (species not specified) (206). Higher supplementation of Spirulina (10%) results in a stronger algal flavor in the bread which was overall, considered satisfactory (204).

In terms of bread rheology, only *C. vulgaris* has reported positive effects on dough rheology and viscoelastic characteristics of wheat flour bread when added at 1% (209) to 3% (210). Higher concentrations (5%) of microalgae resulted in negative effects on dough rheology, bread texture, and flavor (210). Cell wall disruption of *C. vulgaris* prior to incorporation did not significantly alter the viscoelastic characteristics, but improved the firmness of bread, suggesting positive effects of microalgae pre-treatment on bread texture (209).

Adding 1 and 3% Spirulina (*A. fusiformis*) biomass diluted gluten and starch in the dough, affecting the volume of bread (205). Addition of 12% of three different microalgae species *Nannochloropsis gaditana* (*N. gaditana*), *T. chui*, and *C. vulgaris* resulted in weaker dough strength, decreased bread volume, and increased crumb firmness (205). Nevertheless, all of these was improved if the biomass was pre-treated with ethanol (211). Ethanol pre-treatment reduced unpleasant smells and intense colors associated with the microalgae, but it should be noted that it affects overall protein digestibility negatively (211). Among microalgae species, *N. gaditana* combined with ethanol treatment exhibited the best effects for protein fortification of wheat bread (211).

Another study involving 79 sensory panelists revealed that incorporating 1% (w/w) *A. nodosum* into whole meal bread was acceptable (129). In the same study, Hall et al. conducted a crossover trial comparing the post-prandial effects, in which participants who consumed seaweed-enriched bread experienced significantly reduced energy intake (16.4%) in the next meal (129), suggesting that even a low incorporation rate of 1% seaweed in bread can have satiety-promoting effects (Table 1).

Besides nutrient fortification, fortification of seaweed for bioactive purposes has been investigated. Previous research found that adding 4% *P. palmata* protein hydrolysate to wheat bread retained its renin inhibitory bioactivity even after the baking process but did not significantly affect its texture or sensory properties, highlighting a potential for baked products to deliver bioactive protein hydrolysates (212).

##### 5.4.2.2. Gluten-free bread

Algal supplementation has a positive influence on gluten-free bread, as opposed to substitution with plant-based, non-wheat proteins such as peas, potatoes, and zein isolates, which have been demonstrated to impair gluten-aggregation and bread properties (213, 214). Up to 4% Spirulina (*A. platensis*) supplementation in rice flour did not affect or even increase the volume and firmness of the bread (203, 206). But when 5% Spirulina (*A. platensis*) was added into bread flour, a 22% reduction in bread volume and a 113% increase in bread hardness was reported (203).

Two studies independently showed favorable results using *N. gaditana* in gluten-free bread (207, 215). Khemiri et al. (207) compared the effect of *N. gaditana* L2 and *Chlamydomonas* sp. EL5 incorporated into gluten-free bread and reported improved structure, nutritional value, and sensory evaluation (207). The 3% *N. gaditana* L2 bread received the highest score for overall purchase intention, despite the noticeable color changes (207). Qazi et al. compared the effect of adding 4% *T. chui*, *C. vulgaris*, or *N. gaditana* in gluten-free bread (215). Similarly, *N. gaditana* biomass with ethanol treatment produced gluten-free bread with improved technofunctional properties and sensory properties, making it a potential candidate for functional gluten-free bread development (215).

Brown seaweed biomass *A. nodosum*, when included in gluten-free bread ranging from 2 to 4%, resulted in favorable results, such as larger volume, improved texture, and increased antioxidant activity compared to the control bread (216). Further increasing the seaweed biomass resulted in undesirable changes in color, hardness, taste, and bread volume (216).

##### 5.4.2.3. Patisseries

A study conducted by Massoud et al. (217) evaluated the impact of incorporating Spirulina (*A. platensis*) biomass into croissants at concentrations ranging from 0.5 to 1.5%. Results revealed several positive effects of Spirulina (*A. platensis*) fortification such as improved textural and organoleptic properties of the croissants, including increased protein and moisture levels, as well as water-holding capacity, which enhanced their overall quality (217). Notably, the study indicated that optimal sensory results were obtained when Spirulina (*A. platensis*) was incorporated at a concentration of 1% (217).

Khosravi-Darani et al. (218) conducted a study to explore the effects of Spirulina (*A. platensis*) addition on strudels at different levels (0 to 1.5% w/w). The results demonstrated that the enriched strudels had higher protein content and reduced peroxide value, as well as better color stability over a 45-day period. The sensory analysis indicated that the addition of 0.5 and 1% Spirulina (*A. platensis*) resulted in the most preferred texture and color.

Another study by Niccolai et al. (219) investigated the influence of Spirulina (*A. platensis*) biomass (2, 6, and 10% w/w) incorporation in “crostini,” a leavened bakery product commonly consumed in Italy and other parts of Europe. Results showed that the crostini doughs with Spirulina (*A. platensis*) reached a slightly lower, but still appropriate, volume after fermentation compared to the control. Furthermore, crostini fortified with 6 and 10% Spirulina (*A. platensis*) exhibited significantly higher protein content, antioxidant capacity and phenolic content, but slightly lower *in vitro* digestion.

##### 5.4.2.4. Cookies and biscuits

Incorporating 4% Spirulina (*A. platensis*) into biscuits led to increased hardness and crispiness levels, and the highest sensory color scores (220). However, substituting wheat flour with 2% *C. vulgaris* biomass revealed no noticeable impact on the texture of wheat cookies, but resulted in the highest acceptance score. However, when the *C. vulgaris* content was increased to 6%, a significant rise in hardness was observed (221). In 4 species investigated, including Spirulina (*A. platensis*), *C. vulgaris*, *Tetraselmis suecica*, and *P. tricornutum*, 2% microalgal biomass had a significant impact on smell, taste, and overall acceptability (221). This effect was attributed to the presence of sulfuric compounds, diketones, α-ionone, and β-ionone (221). It is noteworthy that the addition of up to 6% *C. vulgaris* without affecting the overall sensorial properties was possible when the biomass was defatted (222). In fact, it was reported that the aroma of the cookies remained acceptable even with a 9% incorporation of defatted biomass (222).

From a nutritional perspective, incorporating algal biomass into cookies and biscuits increased the protein content, phenolic content, and antioxidant potential (220, 221). According to Batista et al., cookies prepared with *C. vulgaris* and *A. platensis* exhibited higher protein content compared to *T. suecica* and *P. tricornutum* (221). No significant difference in *in vitro* digestibility was found between microalgae cookies and the control (221).

#### 5.4.3. Snacks

The dried biomass of *E. gracilis* has been explored for use in various food products for its rich β-glucan polysaccharides, including in grains, grain-based products, breakfast items, granola, and protein bars, as reviewed in (223). Its inclusion in these food items can contribute to their nutritional profile and provide the benefits associated with β-glucan polysaccharides.

Homemade roasted seaweed snacks, prepared from raw dried seaweed sheets, have gained popularity as a healthy, cost-effective alternative to packaged seaweed snacks. These snacks offer a convenient and nutritious option for consumers seeking a homemade, additive-free alternative.

Lucas et al. (224) developed snacks enriched with 2 and 6% Spirulina, which resulted in significant increases in protein content, and increased stability of the snacks during a 30-day storage period.

Da Silva et al. (225) conducted a study to examine the antioxidative properties of Spirulina in the context of snack production. The study compared the intact, hydrolysate, and hydrolyzed peptide fractions (<4 kDa or > 4 kDa) of Spirulina, evaluating their antioxidant potential and thermal stability when added to extruded snacks. The results demonstrated that snacks fortified with 2% bioactive peptides derived from Spirulina exhibited higher antioxidant activity, presenting a highly viable natural option to make functional food.

Incorporating 4% Spirulina (*A. platensis*) biomass into white chocolate significantly enhanced its nutritional value (226). Despite these changes, sensory scores did not show significant differences between nutrient-enriched products and control chocolate (226). These findings highlight the potential of Spirulina (*A. platensis*) biomass as a valuable enrichment ingredient for white chocolate production.

### 5.5. Algal supplementation in liquid food and beverages

Microalgae biomass, such as Spirulina, *Chlorella*, or *Tetraselmis* (all species not specified), when incorporated into a broccoli-based soup at concentrations ranging from 0.5 to 2.0%, have been found to increase viscosity, antioxidant capacity, and phenolic content of the soup (227). The most accepted formulation was the one containing 0.5% *Tetraselmis*, though increasing the percentage resulted in reduced sensorial acceptability compared to the control (227).

Another study involved the incorporation of Spirulina (*A. platensis*) in kefir and found supplementation with 0.05 and 0.1% Spirulina (*A. platensis*) to be most favorable, with these concentrations mildly elevating the protein content from 27 to 37 mg/mL (228). A more pronounced increase in amino acid content was observed in kefir enhanced with 1% Spirulina (*A. platensis*) (228).

A Dutch startup, Ful, has developed a bright blue soda made with Spirulina (species not specified), a blue-green algae, which is marketed as a nutritious and environmentally sustainable product with a reduced carbon footprint and which actively captures CO_2_ into ingestible nutrients (229).

Researchers have measured the antioxidant activity and total phenolic content of various juices containing Spirulina (*A. platensis*), such as apple juice, Japanese quince syrup, and cranberry syrup, and discovered that the resulting products have at least the same antioxidant activity as pure Spirulina (*A. platensis*) and a longer shelf life (230), indicating their potential for juice incorporation.

#### 5.5.1. Milk and dairy products

Algae have been used as an additive for milk and yoghurt to enhance the viability of probiotics (231). Beheshtipour et al. (231) found that adding *C. vulgaris* at concentrations of ≤1.0% resulted in increased acidity and redox potential, without affecting pH levels significantly. However, sensory attributes, including oral texture, mouthfeel, appearance, and non-oral texture, were negatively affected (231). These findings suggest that microalgae addition into milk and dairy products may pose challenges in terms of palatability.

Incorporating microalgae into cheese has positive effects on nutritional values and product outcomes. Mohamed et al. (232) demonstrated that adding *C. vulgaris* at various concentrations (1 to 3% w/w) increased firmness and decreased oil separation indexes of cheese. Tohamy et al. (233) found that increasing *C. vulgaris* (2 to 6% w/w) led to an increase in pH and meltability of the spread cheeses compared to the control sample. In general, all supplementation reported better nutritional attributes in the product (232–234).

Similar conclusions were reached in a recent review article discussing the impact of adding microalgal biomass, such as *C. vulgaris* and *A. platensis*, on the composition, properties, texture, and sensory profile of dairy products like yogurt, cheese, and ice cream (235). The literature review reveals that increasing microalga content decreases yogurt palatability. In ice cream, microalgal biomass reduces melting time and replaces artificial dyes with stable natural ones. Cheese with microalgae has enhanced antioxidant capacity due to high phenolic and carotenoid contents. However, the incorporation of microalgae negatively affects the sensory attributes of these products, a major challenge that requires further research to strike a balance between improved nutrition and sensory quality (235).

Another noteworthy advancement is the use of concentrated microalgae flour to generate lactose-free plant-based milk with a nutritional profile comparable to that of cow’s milk and high-quality EAA (236, 237). In contrast to past efforts that added algal-based ingredients as a supplement or enhancement, this product is a pure algal-based dairy substitute created from microalgal-based protein-rich flour. The protein-rich flour can be modified by altering the ratio of water-soluble microalgae flour, allowing for variations in protein content and enhancing textural and sensory aspects, which has led to the development of microalgae-based vegan ice creams, yoghurts, and cheeses (238).

#### 5.5.2. Beer

The marine species *T. chui*, renowned for its starch accumulation capabilities, has been studied as a potential active ingredient in beer brewing. Incorporating *T. chui* into the brewing process has the potential to influence the flavor profiles of beers as the compounds derived from microalgae can contribute to the complexity and uniqueness of flavors, providing consumers with novel sensory experiences. However, comprehensive studies investigating the specific flavor compounds and their impact on beer characteristics are still limited (239). Notably, one significant advantage of algal supplementation in beer brewing is its potential for carbon capture, which reduces their carbon footprint and contributes to climate change mitigation. Young Henrys, a craft brewery in Sydney, Australia, has been at the forefront of this approach and is credited as the first brewery to use algae for capturing CO_2_ during fermentation (240, 241).

### 5.6. Considerations when incorporating algae into future foods

Algae and its ingredients have demonstrated nutritional-enhancing properties, particularly in boosting protein content and antioxidant activity in food preservation. Multiple studies have found no significant changes in overall acceptability when supplementation was done at low percentages (< 5%) (192, 203, 206, 226), indicating the potential for supplementing into various food types.

However, results on the sensory qualities of greater amounts of algae in supplemented foods are less convincing, primarily due to the strong sensory characteristics that impair the appearance and flavor. In general, studies suggest that incorporating low percentages of algal biomass results in acceptable, and sometimes, preferred products, whereas high percentage incorporation can be undesirable. The optimal balance for carbohydrate-rich foods is struck when supplementing up to 6–7% of algae in biscuits, cookies (even up to 9%, if defatted), and snacks, as well as up to 12.5% for pasta. These percentages have been reported without compromising sensory evaluations, indicating potential for further research in carbohydrate-rich food.

Even if sensory evaluation might be compromised, low percentage of biomass incorporation can enhance textural, organoleptic, and rheological properties, rendering the final products acceptable. Regarding the rheology of baked goods, it is reported that algae fortification above 5% increases the hardness of wheat products (209, 221). Similarly, Sahni et al. reported that defatted *C. vulgaris* rendered flour blends with decreased pasting viscosities, which makes it suitable for cookies, but not for bread (222). Overall, this duality between sensory appeal and techno-functionality underpins the feasibility of incorporating algal biomass into carbohydrate staples such as pastas, noodles, cookies, snacks, and biscuits. For protein-rich food fortification and food innovations, algae improve the nutrition and sensory attributes of meat, seafood, and their corresponding animal-free analogs. Algae’s strong seafood attribute makes it an excellent seasoning and preservation agent in canned seafood. In contrast, adding algal biomass into milk and dairy products changes the texture, color, and taste which may result in less appetizing qualities.

### 5.7. Priorities for future research

Future research should focus on several key areas to fully unlock the potential utilization of microalgal and seaweed proteins for human nutrition. Firstly, optimization of sensory attributes to improve product acceptability remains a critical issue. Consumer’s perceptions of algae-based products are largely shaped by their unique taste and texture, which deviate from traditional food ingredients. Consequently, choosing the right cultivars and/or modifying growth conditions as well as modifying processing methods that fine-tune these sensory attributes are pivotal for enhancing product acceptability.

For instance, research by Caporgno et al. indicates that cultivating *A. protothecoides* under mixotrophic and heterotrophic conditions leads to reduced chlorophyll accumulation, thus generating a biomass with a less intense green hue (184). This is potentially more appealing to consumers who may find vibrant colors off-putting. Additionally, such biomass demonstrated superior emulsion properties compared to its phototrophically-produced counterparts.

From our observations, the continued success of algal supplementation hinges on efforts to customize and calibrate appropriate algal biomass for specific food applications. This entails not just modifying cultivation and processing methods but also selecting the appropriate strains and cultivars. Such tailored usage of algae should target to improve the acceptability of the final product as well as enhance the bioavailability of beneficial components, promoting improved health outcomes. Henceforth, it is imperative to prioritize the study of algal biology to enhance our comprehension of algae metabolism and facilitate advancements in biomass technologies. Subsequent to cultivation, downstream processing methods are able to further refine algae’s organoleptic properties. Numerous studies advocate for advanced processing techniques—such as protein extraction, ethanol treatment, and defatting—to enhance the end-product’s quality over that of crude biomass.

Next, optimization of extraction methods merits further investigation. Simple pre-processing methods like cell disruption can significantly elevate the bioavailability of nutrients, especially in algae strains with hard-to-digest cell walls. Defatting, another pre-processing method, can help remove the undesirable taste of algae. Continued exploration of effective extraction techniques such as ultrasound-assisted, microwave-assisted, and pulsed electric field extraction is necessary to overcome the challenges posed by cell wall polysaccharides. However, it is important to approach such preprocessing with caution, as the disruption of cells might also lead to the loss of some beneficial compounds or increased production cost. Striking the right balance in preprocessing will be an important aspect of future research and development in the field of algal food applications.

Lastly, future research should delve into the spatial and agroecological dimensions of algal farming. Optimizing the nutrient profiles of locally grown algae strains offers significant ecological benefits. A focus on localized cultivation enhances sustainability and allows for tailoring to local ecological conditions. This also streamlines the production process, potentially increasing crop yields and reducing transportation-related emissions. By aligning algae strains with local environmental factors, researchers could unlock avenues for more efficient and environmentally responsible protein production. Such an approach could also stimulate local economies and contribute to community resilience. Therefore, targeted research and planning in the spatial and agroecological aspects of algal farming represent a crucial step toward realizing algae’s full potential as a sustainable and nutritious protein source.

## 6. Conclusion

Taken together, this review has summarized multiple studies that underscore the potential of algae as a viable, nutritious and sustainable source of protein. Approximately 50% of certain microalgae species and around 25% of red and green seaweeds are constituted from proteins with amino acid profiles comparable to those of conventional protein sources. While there are challenges in extracting protein from algae, these do not preclude their applications in food as their co-nutrients (including dietary fiber, bioactive phytonutrients such as polyphenols and carotenoids) provide extra benefits, such as enhanced satiety and lipid profiles, as shown in human trials. Future trials should prioritize studies that assess the safety and protein bioavailability of algal biomass or protein extracts on humans.

The potential of algae as a prospective food source is rooted in its nutritional composition and associated health advantages, as well as its physicochemical attributes that make it suitable for various food-related purposes. The addition of algal proteins in various food products, such as protein-rich plant-based meats, seafood-related products, as well as hybrid meats and seafoods as well as baked goods, can improve the overall acceptability of the final products, although further efforts are needed to increase consumer acceptance and enjoyment of these products.

In conclusion, the incorporation of algae into our food system has great potential but requires further multidisciplinary investigations. Key areas for future research include the scalability of protein and nutrient extraction methods, localizing algal production focusing on life-cycle analyses, cost, and sustainability. Tailored strain selection and optimized cultivation, along with efficient downstream processing, can significantly improve both the desirability and nutritional quality of algae-derived food products.

## Author contributions

JW: Writing – original draft, Writing – review & editing. RT: Writing – original draft, Writing – review & editing. HT: Writing – original draft. SH: Conceptualization, Funding acquisition, Supervision, Writing – review & editing.
