# Supplementary material for: The utility of algae as sources of high value nutritional ingredients, particularly for alternative/complementary proteins to improve human health
Source: Front Nutr. 2023 Oct 13;10:1277343. doi: 10.3389/fnut.2023.1277343 (PMC10613476; doi:10.3389/fnut.2023.1277343)
Supplement: Supplementary file 1 [file Table_1.docx]

**Supplementary Table 1**

**Amino acid compositions (expressed in grams per 100 grams of protein) of various macro- and microalgae, extracted from literatures. Green box indicates amino acids content higher than the FAO recommendation.**

| **Species Name** | **His** | **Ile** | **Leu** | **Lys** | **Met** | **Cys** | **Phe** | **Tyr** | **Thr** | **Trp** | **Val** | **Ala** | **Arg** | **Asp** | **Asn** | **Glu** | **Gln** | **Gly** | **Pro** | **Ser** | **EAA** |
| --- | --- | --- | --- | --- | --- | --- | --- | --- | --- | --- | --- | --- | --- | --- | --- | --- | --- | --- | --- | --- | --- |
| **FAO recommendation (1)** | **1.6** | **3** | **6.1** | **4.8** | **2.3** | | **4.1** | | **2.5** | **0.66** | **4** |  |  |  |  |  |  |  |  |  |  |
| *Alaria esculenta* | 1.4 | 3.1 | 5.5 | 5.2 | 1.3 | ND | 3.6 | 2.9 | 4.7 | ND | 4.9 | 13.3 | 3.4 | 11.9 | ND | 25.9 | ND | 4.6 | 3.4 | 4.8 | 32.6% |
| *Arthrospira platensis* | 1.9 | 4 | 8.7 | 6.3 | 1.7 | 1.7 | 7 | 7 | 4.1 | 1 | 6.9 | 10.2 | 5.4 | 9 | ND | 12.8 | ND | 4.7 | 4.6 | 2.9 | 50.4% |
| *Arthrospira platensis* | 1.8 | 5.9 | 10 | 5.5 | 2.8 | 1 | 5.4 | 5 | 5.4 | 1.4 | 6.8 | 8.1 | 7.2 | 10.2 | ND | 13.7 | ND | 5.6 | 4.1 | ND | 51.1% |
| *Arthrospira platensis* | 2 | 6.6 | 10.6 | 6 | 2.8 | 0.4 | 5.9 | 5.3 | 5.8 | 1.5 | 7.4 | 7.9 | 11.1 | 14.9 | ND | 6.1 | ND | 4.5 | 1.1 | ND | 54.4% |
| *Ascophyllum nodosum* | 2.9 | 5.1 | 8.8 | 5.6 | 1.3 | 6.9 | 6.7 | 3.2 | 5.9 | ND | 6.1 | 6.4 | 4 | 12.3 | ND | 14.7 | ND | 4.8 | ND | 5.3 | 52.5% |
| *Ascophyllum nodosum* | 3.5 | 5.1 | 7.3 | 10.5 | 1.3 | ND | 3.8 | 2.9 | 6 | 0.3 | 6 | 4.8 | 5.4 | 13 | ND | 22.9 | ND | ND | ND | ND | 50.3% |
| *Ascophyllum nodosum* | 1.9 | 5.2 | 9.9 | 5.2 | 1.9 | 0.3 | 5 | 1.9 | 4.7 | ND | 6.9 | 7.7 | 5.5 | 12.7 | ND | 20.4 | ND | 6.3 | ND | 4.4 | 42.9% |
| *Ascophyllum nodosum* | 1.4 | 4.4 | 7.5 | 5.5 | 2 | ND | 5.3 | 2.9 | 5.8 | ND | 6 | 6.9 | 4.4 | 16 | ND | 16.3 | ND | 5.9 | 4.5 | 5.4 | 40.7% |
| *Bifurcaria bifurcata* | 1.8 | 4 | 7.2 | 5.4 | 2.3 | ND | 4.6 | 2.3 | 5 | ND | 5.1 | 11.6 | 4.6 | ND | 11 | 20.7 | ND | 5.4 | 4.3 | 4.8 | 37.7% |
| *Caulerpa lentillifera* | 1.3 | 4.2 | 7.6 | 5.6 | 1.4 | 1 | 3.8 | 2.9 | 5.9 | ND | 6.6 | 8.7 | 3.8 | 10 | ND | 11.1 | ND | 15.4 | 4.4 | 6.2 | 40.3% |
| *Caulerpa lentillifera* | 0.6 | 5 | 8 | 6.6 | ND | ND | 4.9 | 3.9 | 6.4 | ND | 7 | 6.9 | 7 | 11.6 | ND | 14.4 | ND | 6.9 | 4.6 | 6.1 | 42.4% |
| *Caulerpa lentillifera* | 1.5 | 4.7 | 8.7 | 5.3 | 1.6 | 0.6 | 4.5 | 3.2 | 6.3 | ND | 7.6 | 9.1 | 3.9 | 10.1 | ND | 10 | ND | 12.4 | 3.9 | 6.4 | 44.1% |
| *Caulerpa lentillifera* | 1.4 | 5 | 7.7 | 1.2 | 1.6 | ND | 19.6 | 3.3 | 5.7 | ND | 6.1 | 6.8 | 5.6 | 8.2 | ND | 13.2 | ND | 5.1 | 4.2 | 5.4 | 51.5% |
| *Caulerpa racemosa* | 2.4 | 6.1 | 9.6 | 6 | 2.4 | ND | 5.8 | 3.1 | 4.3 | ND | 8.6 | 7.4 | ND | 11.5 | ND | 18.7 | ND | 5.7 | 4.4 | 3.5 | 48.5% |
| *Chlorella pyrenoidosa* | 2 | 3.8 | 9.8 | 5.7 | 3 | 0.1 | 7.3 | ND | 4.1 | ND | 8.7 | 7.1 | 15.4 | 7.7 | ND | 10.8 | ND | 4.8 | 5.4 | 4.2 | 44.5% |
| *Chlorella sp.* | 2.1 | 3.9 | 9.1 | 8.7 | 2.3 | 1.4 | 5.2 | 3.5 | 4.9 | 2.2 | 5.7 | 8.1 | 6.6 | 9.3 | ND | 12 | ND | 6 | 4.9 | 4.2 | 49.0% |
| *Chlorella vulgaris* | 1.5 | 3.4 | 8.5 | 5.4 | 2.5 | 1.5 | 6.2 | 4.4 | 5.5 | 0.2 | 6.9 | 10.9 | 7.4 | 8.6 | ND | 10.4 | ND | 7.2 | 5.1 | 4.4 | 46.0% |
| *Chondrus crispus* | 2.1 | 4.5 | 6.9 | 5.3 | 3.3 | 0.7 | 4.3 | 2.7 | 5.5 | ND | 6.2 | 7.5 | 6.5 | 12 | ND | 12.1 | ND | 5.2 | 5.6 | 5.1 | 43.5% |
| *Chondrus crispus* | 5.6 | 5 | 6.3 | 10.7 | 0.6 | ND | 4.7 | 4.4 | 4.4 | ND | 6.3 | 1.3 | 8.8 | 11.3 | ND | 9.7 | 0.9 | ND | ND | 14.4 | 50.8% |
| *Chondrus crispus* | 1.9 | 4.3 | 7.1 | 7.8 | 1.8 | ND | 5.8 | 3.6 | 5.3 | ND | 6.1 | 6.5 | 8.8 | 11.6 | ND | 12.6 | ND | 5.6 | 5.6 | 5.6 | 43.7% |
| *Chordaria flagelliformis* | 1.8 | 4.3 | 8.1 | 5.9 | 2.4 | ND | 5.1 | 3.1 | 5.6 | ND | 5.9 | 10.3 | 5 | 12.3 | ND | 13.6 | ND | 6.1 | 4.9 | 5.5 | 42.2% |
| *Cladophora rupestris* | 1.4 | 3.6 | 7 | 7.4 | 1.8 | ND | 4.5 | 4.3 | 5.1 | ND | 5.8 | 5.5 | 6.5 | 15.3 | ND | 15.3 | ND | 6.7 | 5.7 | 4.3 | 40.8% |
| *Codium fragile* | 1 | 4.4 | 7.7 | 5.5 | 9.9 | 1.1 | 4.4 | 3.3 | 5.5 | ND | 15.4 | 6.6 | 4.4 | 8.8 | ND | 11 | ND | 5.5 | ND | 5.5 | 58.2% |
| *Dunaliella tertiolecta* | 1.3 | 3.1 | 7.6 | 4.7 | 1.5 | 1.6 | 5 | 3.1 | 4.9 | 1.6 | 5 | 7.3 | 16.1 | 9 | ND | 13 | ND | 6 | 4.3 | 4.9 | 39.4% |
| *Dunaliella salina* | 1.6 | 3.7 | 8.7 | 5.4 | 2.5 | 1.5 | 6.3 | 4.4 | 4.7 | 0.2 | 6.6 | 10 | 7.4 | 8.7 | ND | 11.3 | ND | 7.9 | 4.8 | 4.4 | 45.6% |
| *Dunaliella viridis* | 1.7 | 5 | 9.2 | 5.5 | 3.7 | 0.7 | 7.4 | 6 | 5.7 | ND | 6.4 | 6.5 | 12.9 | 3 | ND | 6 | ND | 6.1 | 8.3 | 5.2 | 51.7% |
| *Eucheuma cottonii* | 0.5 | 4.6 | 6.4 | 2.7 | 1.6 | ND | 36.1 | 1.9 | 4 | ND | 4.9 | 5.9 | 4.9 | 5 | ND | 9.8 | ND | 4.3 | 3.8 | 3.6 | 62.7% |
| *Euglena gracilis* | 2.7 | 4.2 | 8.7 | 7.3 | 2.3 | 1.6 | 4.8 | 4.2 | 5 | 1.9 | 6.7 | 7.4 | 7 | ND | 8.5 | 12 | ND | 5.2 | 6.3 | 4.2 | 49.4% |
| *Fucus lumbricalis* | 1.6 | 4 | 6.8 | 5.6 | 1.9 | ND | 7.3 | 4.2 | 5 | ND | 5.8 | 5.8 | 13.5 | 10 | ND | 11.9 | ND | 5.6 | 5.8 | 5.2 | 42.2% |
| *Fucus serratus* | 1.7 | 4 | 6.8 | 5.5 | 1.9 | ND | 5 | 3.7 | 5.5 | ND | 5.6 | 6.8 | 4.4 | 14 | ND | 19.6 | ND | 5.8 | 4 | 5.6 | 39.7% |
| *Fucus spiralis* | 2.1 | 10.1 | 10.2 | 8.3 | 4.2 | ND | 6.5 | 5.1 | 7.1 | ND | 7.5 | 2.6 | 7.7 | 3.7 | ND | 8 | ND | 4.9 | 4.6 | 7.5 | 61.0% |
| *Fucus spiralis* | 1.8 | 4.6 | 8.1 | 6.4 | 2.4 | ND | 5.7 | 3.7 | 5.5 | ND | 6 | 7.1 | 4.9 | 14.2 | ND | 13.4 | ND | 6 | 4.6 | 5.6 | 44.2% |
| *Fucus spiralis* | 4.4 | 5.2 | ND | 10.2 | 0.6 | ND | 3.3 | ND | 7.4 | 0.3 | 6.1 | 1.9 | 4.1 | 14.3 | ND | 19.8 | 0.3 | ND | ND | 15.2 | 40.3% |
| *Fucus vesiculosus* | 1.6 | 4.2 | 7.3 | 6.8 | 1.8 | 1.7 | 4.6 | 2.7 | 5.2 | ND | 4.9 | 8.3 | 4.6 | ND | 14.1 | 16.7 | ND | 5.5 | 4.8 | 5.3 | 40.8% |
| *Fucus vesiculosus* | 1.7 | 4.4 | 7.9 | 6.3 | 2.2 | ND | 5.1 | 3.1 | 5.6 | ND | 6 | 7.2 | 4.9 | 14.5 | ND | 15 | ND | 5.7 | 4.6 | 5.6 | 42.4% |
| *Gelidium corneum* | 1.8 | 5.5 | 9.7 | 7.3 | 0.6 | ND | 6.1 | 4.2 | 4.2 | ND | 8.5 | 11.5 | ND | 12.1 | ND | 9.7 | ND | 4.8 | 9.1 | 4.8 | 47.9% |
| *Gracilaria chilensis* | 8.5 | 6.2 | 3.1 | 4.7 | 14 | 5.4 | 7.8 | 2.3 | 4.7 | ND | 5.4 | 4.7 | 4.7 | 8.5 | ND | 11.6 | ND | 3.1 | ND | 5.4 | 62.0% |
| *Gracilaria domingensis* | 1.3 | 5.2 | 9.2 | 5.2 | 2.6 | 0.4 | 5.2 | 2.6 | 5.2 | 2.6 | 5.2 | 7.9 | 5.2 | 13.1 | ND | 11.8 | ND | 6.6 | 5.2 | 5.2 | 44.8% |
| *Gracilaria foliifera* | 1.2 | 10.1 | 10.3 | 3.6 | 0.9 | ND | 8 | 1.1 | 1.5 | ND | 12.7 | 12.2 | 8.7 | 6.8 | ND | 4.6 | ND | 2.7 | 12.2 | 3.4 | 49.4% |
| *Gracilaria gracilis* | 0.7 | 8.5 | 7 | 5.9 | 0.7 | 1.5 | 6.3 | 4.8 | 6.3 | 3.3 | 11.4 | 7 | 4.8 | 9.6 | ND | 8.8 | ND | 4 | 3.7 | 5.9 | 56.3% |
| *Gracilaria gracilis* | 0.6 | 5 | 6.7 | 7.3 | 1.7 | ND | 5 | 3.4 | 5.6 | ND | 5.6 | 6.7 | 7.8 | 11.7 | ND | 14 | ND | 7.3 | 5 | 6.7 | 40.9% |
| *Gracilaria vermiculophylla* | 1.1 | 5.5 | 8.5 | 5.4 | 1.3 | ND | 5.8 | 3.3 | 5.8 | 0.4 | 6.4 | 7.6 | 7.9 | 11.9 | ND | 11.7 | ND | 6.4 | 4.5 | 6.3 | 43.6% |
| *Halidrys siliquosa* | 1.5 | 4 | 6.8 | 5.4 | 2.1 | ND | 4.5 | 3.1 | 5.2 | ND | 5.9 | 6.5 | 4.7 | 11.5 | ND | 25 | ND | 4.9 | 4 | 4.9 | 38.5% |
| *Himanthalia elongata* | 3.7 | 4.2 | 3.8 | 6 | 3.6 | 5.8 | 4.2 | 2.6 | 6 | ND | 7.9 | 6.1 | 5.6 | 11 | ND | 13.9 | ND | 5.5 | 4.7 | 5.1 | 47.9% |
| *Himanthalia elongata* | 1.9 | 4.3 | 7.6 | 7 | 1.9 | ND | 4.9 | 3.7 | 5.9 | ND | 6.1 | 6.9 | 5.1 | 14.2 | ND | 14.4 | ND | 6 | 4.1 | 6 | 43.3% |
| *Hizikia fusiforme* | 2.9 | 4.4 | 7.4 | 3.4 | 1.8 | 1 | 5.1 | 3.1 | 4.5 | 0.4 | 5.4 | 4.7 | 5 | 10 | ND | 20.6 | ND | 5.3 | 4.2 | 4.1 | 42.2% |
| *Kappaphycus alvarezii* | ND | 6.1 | 9.4 | 4.4 | 2.2 | ND | 6.2 | 2.2 | 5.4 | ND | 7.3 | 8.3 | 4.7 | 13.5 | ND | 13.3 | ND | 5.9 | 4.7 | 6.4 | 43.2% |
| *Kappaphycus alvarezii* | 1.1 | 5.5 | 8.4 | 3.7 | 1.8 | ND | 6.3 | 2.4 | 5 | ND | 6.3 | 8.4 | 4.2 | 11.6 | ND | 11.9 | ND | 6.1 | 10.3 | 6.9 | 40.5% |
| *Laminaria digitata* | 1.8 | 3.6 | 6.6 | 5 | 2.1 | ND | 4.6 | 2.9 | 5.4 | ND | 5.1 | 16.6 | 4.8 | 11.9 | ND | 14.7 | ND | 5.3 | 4.9 | 4.7 | 37.1% |
| *Laurencia filiformis* | 1.8 | 4.4 | 7.1 | 8.8 | 2.7 | 0.9 | 4.4 | 5.3 | 5.3 | 1.8 | 4.4 | 6.2 | 5.3 | 13.3 | ND | 12.4 | ND | 6.2 | 4.4 | 5.3 | 46.9% |
| *Laurencia intricata* | 1.5 | 4.5 | 7.4 | 7.4 | 1.5 | 0.6 | 4.5 | 4.5 | 5.9 | 1.5 | 4.5 | 7.4 | 3 | 14.8 | ND | 13.4 | ND | 7.4 | 4.5 | 5.9 | 43.7% |
| *Laminaria japonica* | 1.8 | 5.2 | 8.8 | 5.5 | 1.9 | 8.3 | 5.8 | 3.3 | 5.6 | ND | 6.3 | 6.5 | 4.4 | 11.8 | ND | 14.6 | ND | 5.1 | ND | 5.3 | 52.4% |
| *Laminaria japonica* | 4.3 | 4.7 | 8 | 8.6 | 2.7 | 3.5 | 5.1 | 3.1 | 6.1 | 0.3 | 10.8 | 8.1 | 5.3 | 8.4 | ND | 7 | ND | 6 | 3.4 | 4.4 | 57.3% |
| *Laminaria japonica* | 2.5 | 5.1 | 9.4 | 6.3 | 0.9 | 8.3 | 6 | 3.1 | 6 | ND | 6.9 | 6.3 | 5.7 | 11.2 | ND | 12.7 | ND | 4.3 | ND | 5.5 | 54.4% |
| *Laminaria japonica* | 1.2 | 3.5 | 5.5 | 0.5 | 1.8 | 0.5 | 2.5 | 1.8 | 4.8 | 0.5 | 6 | 9 | 1.8 | 7.8 | ND | 36.6 | ND | 6 | 4.8 | 5.5 | 28.6% |
| *Laminaria japonica* | 1.6 | 2.9 | 5.3 | 2.9 | 1.6 | 0.6 | 3.3 | 1.8 | 4.6 | 0.2 | 4.2 | 8.4 | 2.5 | 22.1 | ND | 28.6 | ND | 3.3 | 2.9 | 3.4 | 28.9% |
| *Laminaria sp.* | 2.5 | 3.1 | 5.7 | 4.5 | 1 | 1.4 | 3.7 | 2 | 4 | 0.6 | 4.4 | 6.6 | 3.8 | 14.4 | ND | 27.5 | ND | 4.6 | 3.6 | 3.8 | 33.8% |
| *Lessonia flavicans* | 1.5 | 3.3 | 6.8 | 4.8 | 0.9 | 1.6 | 4.1 | 4.5 | 8.3 | ND | 6.5 | 9.4 | 2.9 | 17.3 | ND | 17.8 | ND | 4.5 | ND | 6.1 | 42.2% |
| *Lessonia nigrescens* | 1.7 | 4.1 | 7.8 | 7.3 | 2 | 0.5 | 4.7 | 2.9 | 6.1 | ND | 6.8 | 10.7 | 4.2 | 13.9 | ND | 15.6 | ND | 6.1 | ND | 5.6 | 43.9% |
| *Macrocystis pyrifera* | 1.4 | 3.6 | 7.3 | 5.5 | 1.2 | 1.9 | 4.1 | 3.9 | 8 | ND | 6.6 | 7.1 | 4.1 | 16.9 | ND | 17.3 | ND | 5 | ND | 6.1 | 43.5% |
| *Mastocarpus stellatus* | 2.1 | 3.8 | 6.5 | 8.4 | 1.7 | ND | 5.8 | 5.4 | 3.7 | ND | 5.5 | 6.1 | 7.9 | 12.5 | ND | 11.2 | ND | 8.7 | 5.1 | 5.5 | 42.9% |
| *Nannochloropsis granulata* | 1.9 | 4.6 | 9 | 6.9 | 2.9 | 1.3 | 5.1 | 3.4 | 4.4 | 2.3 | 5.8 | 5.8 | 6 | 9.3 | ND | 11.5 | ND | 6.1 | 9.1 | 4.6 | 47.6% |
| *Nannochloropsis oculata* | 2.1 | 4.8 | 7.9 | 6.2 | 1.6 | 0.4 | 6.3 | 4.2 | 5.6 | 1.6 | 6.6 | 7.5 | 7.4 | 7.7 | ND | 10.2 | ND | 5.6 | 9.4 | 5.1 | 47.2% |
| *Nannochloropsis spp* | 2.3 | 5 | 9.2 | 6.4 | 2.2 | 0.8 | 5.2 | 3.4 | 5.3 | 2.4 | 4.7 | 7.4 | 6 | 9.5 | ND | 12.5 | ND | 6 | 6.9 | 4.8 | 46.9% |
| *Nostoc sp.* | 1.9 | 3.5 | 8.9 | 6.1 | 2.1 | 1.5 | 6.8 | 6.5 | 5 | 1 | 6.8 | 9.4 | 5.8 | 8.7 | ND | 11.7 | ND | 6.2 | 5 | 3 | 50.2% |
| *Osmundea pinnatifida* | 9.5 | 5.4 | 6.4 | 7.7 | 1.3 | ND | 3.9 | 4.4 | 7 | ND | 6.7 | 2.8 | 6.2 | 12.4 | ND | 11.6 | 0.3 | 0.8 | ND | 10.1 | 54.2% |
| *Osmundea pinnatifida* | 1.1 | 19.4 | ND | 3.2 | 2.2 | ND | 2.6 | 2.3 | 6.7 | ND | 2.6 | 0.8 | 4.3 | 16 | ND | 14.2 | ND | 2.9 | 18.5 | 3.2 | 40.1% |
| *Palmaria palmata* | 1.7 | 4.3 | 7.2 | 5.7 | 2 | 2.5 | 4.7 | 2.7 | 4.1 | 0.7 | 5.4 | 8.8 | 5.6 | 9.7 | ND | 14.9 | ND | 5.8 | 7.9 | 6.2 | 41.0% |
| *Palmaria palmata* | 1 | 4.3 | 7.7 | 4.6 | 4.3 | ND | 5.8 | 4 | 2.8 | 2.6 | 5.9 | 7.7 | 5.8 | 16 | ND | 9.8 | ND | 10.8 | 3.6 | 3.5 | 42.9% |
| *Palmaria palmata* | 4.6 | 3.6 | 5.9 | 5.6 | 2.7 | 2.1 | 3.8 | 0.5 | 4.7 | ND | 6.1 | 6.3 | 6 | 10.2 | ND | 15.5 | ND | 5.8 | 4.4 | 5 | 42.7% |
| *Palmaria palmata* | 1.6 | 4 | 7 | 7.7 | 2.2 | ND | 5 | 3.4 | 5.3 | ND | 6.6 | 7.6 | 6.8 | 12.5 | ND | 12.3 | ND | 6.5 | 5.5 | 6 | 42.8% |
| *Pelvetia canaliculata* | 1.6 | 4.3 | 8 | 5.7 | 2.3 | ND | 5.3 | 2.6 | 5.4 | ND | 6 | 7.5 | 4.6 | 12.3 | ND | 18.7 | ND | 5.7 | 4.4 | 5.5 | 41.2% |
| *Pleurochrysis carterae* | 1.6 | 3.6 | 8.6 | 6.3 | 2.1 | 1.8 | 6.6 | 6.6 | 4.9 | 1 | 6.5 | 9.9 | 5.9 | 7.9 | ND | 13.1 | ND | 6.1 | 4.4 | 3 | 49.6% |
| *Porphyra columbina* | 1.3 | 2.7 | 7.4 | 6 | 1.7 | 1.9 | 3.7 | 2.5 | 5.9 | 0.6 | 5.8 | 12.5 | 6.2 | 12.2 | ND | 10.5 | ND | 8.9 | 4 | 6.2 | 39.5% |
| *Porphyra dioica* | 2.2 | 4.1 | 8.1 | 8.1 | 1.8 | ND | 4.1 | 3.7 | 4.4 | ND | 4.4 | 11.1 | 8.5 | 12.2 | ND | 11.4 | ND | 6.6 | 3.3 | 5.9 | 40.9% |
| *Porphyra dioica* | 2.3 | 3.9 | 7.7 | 7.8 | 1.9 | ND | 4.1 | 3.5 | 5.2 | 0.5 | 6.4 | 10.6 | 8.3 | 11.3 | ND | 11 | ND | 6.4 | 3.4 | 5.7 | 43.3% |
| *Porphyra dioica* | 1.4 | 4.1 | 8 | 6.5 | 1.3 | ND | 4.7 | 4.2 | 5.9 | ND | 6.8 | 11.4 | 6.9 | 10.7 | ND | 10.5 | ND | 7 | 5 | 5.7 | 42.9% |
| *Porphyra dioica* | 1 | 4 | 8.2 | 5.7 | 1.1 | ND | 4.5 | 3 | 6 | 0.3 | 6 | 11.4 | 7.1 | 11.8 | ND | 11.1 | ND | 8.2 | 4.5 | 5.9 | 39.9% |
| *Porphyra purpurea* | 1.4 | 3.7 | 7.7 | 6.2 | 1.9 | ND | 4 | 3.3 | 5.8 | ND | 6.7 | 13 | 6.3 | 10.9 | ND | 12.6 | ND | 6.2 | 4.8 | 5.4 | 40.7% |
| *Porphyra purpurea* | 2.6 | 4.1 | 6.4 | 2.8 | 1.6 | 0.5 | 9.4 | 3.5 | 6 | ND | 5.8 | 9.6 | 10.8 | 7.9 | ND | 10 | ND | 9 | 4.6 | 5.5 | 42.7% |
| *Porphyra sp.* | 3 | 3.6 | 6.4 | 5.7 | 2.1 | 1.4 | 3.9 | 4 | 6.2 | 0.8 | 6.1 | 7.2 | 6.9 | 9.9 | ND | 11.9 | ND | 6 | 4.1 | 4.7 | 46.0% |
| *Porphyra sp.* | 3 | 4.1 | 7.4 | 6.5 | 2.1 | 1.6 | 4.4 | 4 | 6.5 | 0.9 | 5.6 | 5.3 | 7.4 | 10.3 | ND | 11.6 | ND | 5.1 | 4.5 | 6.1 | 47.8% |
| *Porphyra sp.* | 3.2 | 4 | 7.2 | 6.3 | 2.2 | 1.6 | 4.3 | 4.2 | 6.5 | ND | 6.3 | 7.6 | 7.2 | 10.4 | ND | 12.5 | ND | 6.2 | 4.4 | 6 | 45.8% |
| *Porphyra tenera* | 1.5 | 4.3 | 9.4 | 4.9 | 1.2 | ND | 4.2 | 2.6 | 4.3 | 1.4 | 6.9 | 8 | 17.8 | 7.6 | ND | 7.8 | ND | 7.8 | 6.9 | 3.1 | 40.8% |
| *Porphyra umbilicalis* | 2.8 | 3.7 | 7.5 | 7 | 2.1 | ND | 4 | 3.8 | 5.2 | 0.6 | 5.9 | 10.2 | 7.8 | 11.6 | ND | 11.2 | ND | 7.2 | 3.8 | 5.8 | 42.5% |
| *Porphyra umbilicalis* | 1.4 | 3.7 | 7.5 | 7.2 | 1.2 | ND | 4.6 | 3.5 | 6 | ND | 6.9 | 10.9 | 6.6 | 12 | ND | 11.5 | ND | 6.7 | 4.9 | 5.4 | 42.0% |
| *Porphyra umbilicalis* | 0.9 | 4.3 | 8.3 | 6 | 1.2 | ND | 4.4 | 2.8 | 6.3 | 0.4 | 6.6 | 11.3 | 7.4 | 12.3 | ND | 10.9 | ND | 7 | 4.4 | 5.5 | 41.2% |
| *Porphyridium cruentum* | 1.4 | 6.6 | 7.3 | 6.9 | 3.5 | 0.4 | 6.3 | 5.5 | 7.8 | ND | 3.1 | 8.3 | ND | 14 | ND | 10.2 | ND | 8.6 | ND | 10.1 | 48.8% |
| *Porphyridium cruentum* | 1 | 6.5 | 8.9 | 6.8 | 2.9 | 0.3 | 5 | 4.5 | 5.4 | ND | 3.8 | 12.7 | ND | 13 | ND | 11 | ND | 9.3 | ND | 9.1 | 45.0% |
| *Pyropia columbina* | 1.2 | 2.7 | 7.4 | 6 | 1.6 | 1.9 | 3.7 | 2.5 | 5.9 | 0.6 | 5.8 | 12.6 | 6.1 | 12.3 | ND | 10.6 | ND | 8.9 | 3.9 | 6.1 | 39.4% |
| *Saccharina japonica* | 4.8 | 4.5 | 6.3 | 6.1 | 3 | 1.5 | ND | 2.2 | 6.5 | ND | 6.8 | 7.7 | 6.2 | 15.7 | ND | 14.9 | ND | 7.8 | ND | 6 | 41.7% |
| *Saccharina latissima* | 1.6 | 4.4 | 7.9 | 5.9 | 2.4 | ND | 5.2 | 3.1 | 5.3 | ND | 6 | 11 | 4.8 | 13.4 | ND | 13.8 | ND | 5.6 | 4.5 | 5 | 41.8% |
| *Sargassum fusiforme* | 1.3 | 4.1 | 6.7 | 1.2 | 5.1 | 5.7 | 2 | 2.4 | 8.7 | ND | 5.3 | 5.8 | 12.2 | 10.3 | ND | 7.4 | ND | 6.1 | 7.1 | 8.4 | 42.6% |
| *Sargassum fusiformis* | 3 | 4.6 | 7.7 | 3.6 | 1.8 | 1 | 5.3 | 3.2 | 4.7 | 0.8 | 5.6 | 5 | 5.2 | 10.5 | ND | 21.5 | ND | 5.5 | 4.4 | 6.5 | 41.3% |
| *Sargassum muticum* | 2 | 5.8 | 10.4 | 6.7 | 2.2 | 0.2 | 6 | 3.2 | 5.5 | ND | 7.3 | 7.8 | 6 | 11.9 | ND | 13.6 | ND | 6.5 | ND | 5 | 49.3% |
| *Sargassum muticum* | 2.4 | 5.1 | 9.8 | 6.1 | 1.9 | 0.2 | 5.4 | 3.1 | 5 | ND | 6.8 | 10 | 6 | 12.3 | ND | 14.6 | ND | 6.6 | ND | 4.7 | 45.8% |
| *Sargassum polycystum* | 0.3 | 3.8 | 6.1 | 2.8 | 1.6 | ND | 39.7 | 1.6 | 3.4 | ND | 4.1 | 5.5 | 3.8 | 5.8 | ND | 10.5 | ND | 4.2 | 3.3 | 3.4 | 63.5% |
| *Sargassum maclurei* | 1.4 | 4.1 | 6.9 | 4.6 | 1.4 | 3.9 | 4.5 | 2.3 | 4 | 2.6 | ND | 8.8 | 4.2 | 9.1 | ND | 33.1 | ND | 4.7 | 4.3 | ND | 35.7% |
| *Spirulina* | 1.9 | 5.8 | 9.3 | 4.9 | 2.5 | 0.9 | 4.8 | 4.7 | 5.3 | 1.7 | 6.4 | 8 | 6.9 | 9.7 | ND | 13 | ND | 5.1 | 4 | 5.2 | 48.2% |
| *Tetraselmis chuii* | 1.8 | 3.5 | 7.4 | 5.6 | 1.9 | 0.6 | 5.3 | 3.7 | 4.2 | 3.7 | 5.7 | 6.7 | 13.3 | 9.3 | ND | 12.3 | ND | 5.8 | 5 | 4.2 | 43.4% |
| *Tetraselmis suecica* | 1.8 | 3.5 | 7.9 | 5.9 | 2.3 | 0.7 | 5.8 | 3.8 | 4.1 | 3.8 | 5.6 | 6.8 | 13.1 | 8.8 | ND | 11.1 | ND | 5.8 | 4.7 | 4.6 | 45.2% |
| *Ulva armoricana* | 1.7 | 4.1 | 5.9 | 5.2 | 2.1 | ND | 6.3 | 4.3 | 4.7 | ND | 4.8 | 6.5 | 6.8 | 10.3 | ND | 18.3 | ND | 7.2 | 6 | 5.6 | 39.2% |
| *Ulva capensis* | 1.8 | 3.6 | 7 | 3.8 | 1.6 | ND | 4.1 | 2.1 | 5.2 | ND | 6.5 | 12.2 | 3.4 | 17.8 | ND | 11.3 | ND | 9.1 | 3.7 | 6.6 | 35.8% |
| *Ulva fasciata* | 1.4 | 5 | 9.3 | 7.9 | 0.9 | ND | 6.1 | 4 | 3.1 | ND | 8.5 | 10.9 | ND | 11 | ND | 15.5 | ND | 6.9 | 5.1 | 3.1 | 46.8% |
| *Ulva intestinalis* | 1.3 | 4 | 7.3 | 5.4 | 1.8 | ND | 4.9 | 2.5 | 5.8 | ND | 6.5 | 9.2 | 5.2 | 14.6 | ND | 13.2 | ND | 5.9 | 7.3 | 5 | 39.5% |
| *Ulva lactuca* | 1.8 | 4.2 | 8 | 5.5 | 2.2 | ND | 5.6 | 3.5 | 5.5 | ND | 6.4 | 8.4 | 6.4 | 12.1 | ND | 13.5 | ND | 6.4 | 4.7 | 5.5 | 42.8% |
| *Ulva lactuca* | 1.9 | 3.8 | 7 | 4.4 | 1.7 | 0.4 | 4.1 | 2.2 | 4.9 | ND | 6.4 | 14.7 | 3.7 | 12.8 | ND | 9.3 | ND | 11.1 | 5.5 | 6.1 | 36.8% |
| *Ulva pertusa* | 5 | 4.3 | 8.6 | 5.6 | 2 | ND | 4.8 | 1.7 | 3.8 | 0.4 | 6.1 | 7.6 | 18.5 | 8.1 | ND | 8.6 | ND | 6.4 | 5 | 3.7 | 42.2% |
| *Ulva rigida* | 3.1 | 4.6 | 8.2 | 4.9 | 2 | ND | 6 | 3.4 | 5.1 | 0.9 | 6 | 8.8 | 6.3 | 12.5 | ND | 9.8 | ND | 6.9 | 4.6 | 5.8 | 44.7% |
| *Ulva rigida* | 1.6 | 3.5 | 5.8 | 4.1 | 1.7 | 1.2 | 3.7 | 2.5 | 5.6 | ND | 6.3 | 13.7 | 5.1 | 14.5 | ND | 10.5 | ND | 8.7 | 4.8 | 6.8 | 36.0% |
| *Ulva rigida* | 3 | 4.6 | 8.2 | 4.9 | 1.4 | ND | 6 | 3.4 | 5 | 0.4 | 7.1 | 8.8 | 6.3 | 13.1 | ND | 9.9 | ND | 6.3 | 4.6 | 5.8 | 44.5% |
| *Ulva sp.* | 6.7 | 5.3 | 7.1 | 6.5 | 1.2 | ND | 4.9 | 3.9 | 4.1 | 0.2 | 7.1 | 2.2 | 7.5 | 12.4 | ND | 10.6 | 0.4 | 0.4 | ND | 15.9 | 48.8% |
| *Ulva sp.* | 0.8 | 4.2 | 8.4 | 5.9 | 5.9 | ND | 10.1 | 3.4 | 6.7 | ND | 2.5 | 9.2 | 4.2 | 12.6 | ND | 12.6 | ND | 6.7 | ND | 6.7 | 47.9% |
| *Undaria pinnatifida* | 2.4 | 5.4 | 10.4 | 7.1 | 3.1 | 0.2 | 5.7 | 3.4 | 5 | ND | 7.4 | 8.9 | 6 | 11.1 | ND | 12.7 | ND | 6.4 | ND | 4.7 | 50.2% |
| *Undaria pinnatifida* | 2.9 | 4.8 | 8.7 | 6.6 | 2 | 1.1 | 5.5 | 3.4 | 5.2 | ND | 6.1 | 5.5 | 6.1 | 10.2 | ND | 17 | ND | 6 | 4.2 | 4.7 | 46.3% |
| *Undaria pinnatifida* | 2.8 | 5.2 | 9.5 | 7.3 | 0.5 | 7.9 | 6.3 | 3.2 | 5.4 | ND | 6.5 | 6.3 | 5.3 | 10.9 | ND | 12.7 | ND | 5 | ND | 5.2 | 54.6% |
| *Undaria pinnatifida* | 3.2 | 4.6 | 6.9 | 6.4 | 1.6 | ND | 3.9 | 3.4 | 5.5 | ND | 5.7 | 7.8 | 6.2 | 9.9 | ND | 17.5 | ND | 0.5 | ND | 13.3 | 42.7% |
| *Undaria pinnatifida* | 1.9 | 5.7 | 9.8 | 4.4 | 0.1 | 0.3 | 5.4 | 2.3 | 3.3 | ND | 6.6 | 11 | 10 | 8.5 | ND | 13.6 | ND | 7.4 | 5 | 4.7 | 39.8% |
| *Undaria pinnatifida* | 1.1 | 2.7 | 5.1 | 4.1 | 1.4 | 0.4 | 2.9 | 1.7 | 3.2 | ND | 4.5 | 38.1 | 3.8 | 8.9 | ND | 12.3 | ND | 6.3 | ND | 3.4 | 27.1% |
| Eggs | 4.6 | 5.4 | 7 | 8.7 | 3.5 | ND | 4.6 | 2 | 3.4 | 1.1 | 6.1 | 7.6 | 13.2 | 7 | ND | 11.2 | ND | 3.8 | 3.2 | 7.7 | 46.4% |
| Eggs | 2.9 | 7.9 | 8.4 | 6.3 | 3.8 | 2.7 | 6.9 | 5 | 6 | ND | 8.6 | ND | ND | 13.1 | ND | 15.1 | ND | 5 | ND | 8.2 | 58.6% |
| Eggs | 5.4 | 6.3 | 8.2 | 10.1 | 4.1 | ND | 5.4 | 2.4 | 3.9 | ND | 7.1 | 8.8 | ND | 8.2 | ND | 13 | ND | 4.5 | 3.7 | 8.9 | 52.9% |
| Soybean | 2.8 | 5.7 | 8.3 | 6.9 | 1.4 | 2 | 5.4 | 4 | 4.3 | 1.5 | 5.7 | 5.4 | 8 | 1.4 | ND | 20.5 | ND | 4.8 | 5.7 | 6.2 | 48.0% |
| Soybean | 6.5 | 5.6 | 11.4 | 10.1 | 2 | 1.6 | 3.7 | 3.8 | 6.2 | 2.7 | 7 | ND | 21.1 | 7.9 | ND | 10.2 | ND | ND | ND | ND | 60.7% |
| Soybean | 6.2 | 5.5 | 11.2 | 10 | 2.2 | 2 | 3.7 | 4 | 6.2 | ND | 6.9 | 2 | 21.5 | 8.3 | ND | 10.3 | ND | ND | ND | ND | 57.9% |
| Soybean | 2.5 | 4.7 | 9.4 | 5.2 | 2 | 0.1 | 5.4 | ND | 3.9 | ND | 8 | 4.4 | 14.8 | 9.7 | ND | 15.7 | ND | 4.1 | 4.9 | 5.2 | 41.2% |

His: Histidine; Ile: Isoleucine; Leu: Leucine; Lys: Lysine; Met: Methionine; Cys: Cysteine; Phe: Phenylalanine; Tyr: Tyrosine; Thr: Threonine; Trp: Tryptophan; Val: Valine; Ala: Alanine; Arg: Arginine; Asp: Aspartic Acid; Asn: Asparagine; Glu: Glutamic Acid; Gln: Glutamine; Gly: Glycine; Pro: Proline; Ser: Serine; EAA: Essential Amino Acids; ND: Not determined.

**References**

1. Food and Agriculture Organization. Dietary Protein Quality Evaluation in Human Nutrition: Report of an FAO Expert Consultation, 31 March-2 April, 2011, Auckland, New Zealand. FAO Food Nutr Pap. 2013;92:1-66.

2. Biancarosa I, Espe M, Bruckner C, Heesch S, Liland N, Waagbø R, et al. Amino acid composition, protein content, and nitrogen-to-protein conversion factors of 21 seaweed species from Norwegian waters. Journal of Applied Phycology. 2017;29:1001-9.

3. Machado M, Machado S, Pimentel FB, Freitas V, Alves RC, Oliveira MBP. Amino acid profile and protein quality assessment of macroalgae produced in an integrated multi-trophic aquaculture system. Foods. 2020;9(10):1382.

4. Dawczynski C, Schubert R, Jahreis G. Amino acids, fatty acids, and dietary fibre in edible seaweed products. Food chemistry. 2007;103(3):891-9.

5. Liu Y, Wang Z, Lin Z, Li C, Zhu Y, Li D. Analysis of Nutritional Components and Physicochemical Properties of Different Parts of Sargassum fusiforme. MODERN FOOD SCIENCE & TECHNOLOGY. 2022;38(1):216-23.

6. Magdugo RP, Terme N, Lang M, Pliego-Cortés H, Marty C, Hurtado AQ, et al. An analysis of the nutritional and health values of Caulerpa racemosa (Forsskål) and Ulva fasciata (Delile)—Two chlorophyta collected from the Philippines. Molecules. 2020;25(12):2901.

7. García-Vaquero M, López-Alonso M, Hayes M. Assessment of the functional properties of protein extracted from the brown seaweed Himanthalia elongata (Linnaeus) SF Gray. Food Research International. 2017;99:971-8.

8. Osman NAR, Abdo B, Mohamed SE-T. Assessment of the nutritional value and native agar content of the red alga Gracilaria foliifera (Forsskal) Borgesen from the Red Sea coast of Sudan. J Algal Biomass Utln. 2017;8:48-63.

9. Paterson S, Gómez-Cortés P, De La Fuente MA, Hernández-Ledesma B. Bioactivity and Digestibility of Microalgae Tetraselmis sp. and Nannochloropsis sp. as Basis of Their Potential as Novel Functional Foods. Nutrients. 2023;15(2):477.

10. Cian RE, Fajardo MA, Alaiz M, Vioque J, González RJ, Drago SR. Chemical composition, nutritional and antioxidant properties of the red edible seaweed Porphyra columbina. International Journal of Food Sciences and Nutrition. 2014;65(3):299-305.

11. Zhang M, Ma Y, Che X, Huang Z, Chen P, Xia G, et al. Comparative analysis of nutrient composition of Caulerpa lentillifera from different regions. Journal of Ocean University of China. 2020;19:439-45.

12. Kim Y-S, Kang C-O, Kim M-H, Cha W-S, Shin H-J. Contents of water extract for Laminaria japonica and its antioxidant activity. KSBB Journal. 2011;26(2):112-8.

13. Chen F, Qian J, He Y, Leng Y, Zhou W. Could Chlorella pyrenoidosa be exploited as an alternative nutrition source in aquaculture feed? A study on the nutritional values and anti-nutritional factors. Frontiers in Nutrition. 2022;9.

14. Bombo G, Cristofoli NL, Santos TF, Schüler L, Maia IB, Pereira H, et al. Dunaliella viridis TAV01: A Halotolerant, Protein-Rich Microalga from the Algarve Coast. Applied Sciences. 2023;13(4):2146.

15. Meng W, Mu T, Sun H, Garcia-Vaquero M. Evaluation of the chemical composition and nutritional potential of brown macroalgae commercialised in China. Algal Research. 2022;64:102683.

16. Saravana PS, Choi JH, Park YB, Woo HC, Chun BS. Evaluation of the chemical composition of brown seaweed (Saccharina japonica) hydrolysate by pressurized hot water extraction. Algal Research. 2016;13:246-54.

17. Safi C, Charton M, Pignolet O, Pontalier P-Y, Vaca-Garcia C. Evaluation of the protein quality of Porphyridium cruentum. Journal of Applied Phycology. 2013;25(2):497-501.

18. Bashir S, Sharif MK, Butt MS, Shahid M. Functional Properties and Amino acid Profile of Spirulina platensis Protein Isolates. Pakistan Journal of Scientific & Industrial Research. 2016;59(1):12-9.

19. Hernández H, Nunes MC, Prista C, Raymundo A. Innovative and Healthier Dairy Products through the Addition of Microalgae: A Review. Foods. 2022;11(5):755.

20. Gressler V, Yokoya NS, Fujii MT, Colepicolo P, Mancini Filho J, Torres RP, et al. Lipid, fatty acid, protein, amino acid and ash contents in four Brazilian red algae species. Food chemistry. 2010;120(2):585-90.

21. Ahmed E, Suzuki K, Nishida T. Micro- and Macro-Algae Combination as a Novel Alternative Ruminant Feed with Methane-Mitigation Potential. Animals. 2023;13(5):796.

22. Andreeva A, Budenkova E, Babich O, Sukhikh S, Ulrikh E, Ivanova S, et al. Production, Purification, and Study of the Amino Acid Composition of Microalgae Proteins. Molecules. 2021;26(9):2767.

23. Shuuluka D, Bolton JJ, Anderson RJ. Protein content, amino acid composition and nitrogen-to-protein conversion factors of Ulva rigida and Ulva capensis from natural populations and Ulva lactuca from an aquaculture system, in South Africa. Journal of applied phycology. 2013;25:677-85.

24. Xiren G, Aminah A. Proximate composition and total amino acid composition of Kappaphycus alvarezii found in the waters of Langkawi and Sabah, Malaysia. International Food Research Journal. 2017;24(3):1255.

25. Echave J, Fraga-Corral M, Garcia-Perez P, Popović-Djordjević J, H. Avdović E, Radulović M, et al. Seaweed protein hydrolysates and bioactive peptides: Extraction, purification, and applications. Marine Drugs. 2021;19(9):500.

26. Fleurence J. Seaweed proteins: biochemical, nutritional aspects and potential uses. Trends in food science & technology. 1999;10(1):25-8.

27. Echave J, Otero P, Garcia-Oliveira P, Munekata PE, Pateiro M, Lorenzo JM, et al. Seaweed-derived proteins and peptides: promising marine bioactives. Antioxidants. 2022;11(1):176.
